# Causality of metabolites and metabolic pathways on cholestatic liver diseases: a Mendelian randomization study

**DOI:** 10.3389/fmed.2024.1395526

**Published:** 2024-07-02

**Authors:** Zhengxiao Wei, Yingfen Liu, Qingqing Xiong, Xue Mei, Jinghong Li, Zhangjun Wu

**Affiliations:** ^1^Department of Clinical Laboratory, Public Health Clinical Center of Chengdu, Chengdu, China; ^2^Department of Science and Education Division, Public Health Clinical Center of Chengdu, Chengdu, China; ^3^Department of Infectious Diseases, Public Health Clinical Center of Chengdu, Chengdu, China

**Keywords:** blood metabolites, chloestatic liver diseases, Mendelian randomization, primary biliary cholangitis, primary sclerosing cholangitis, metabolic pathway

## Abstract

**Background and Aims:**

Blood metabolite abnormalities have revealed an association with cholestatic liver diseases (CLDs), while the underlying metabolic mechanisms have remained sluggish yet. Accordingly, the present evaluation aims to investigate the causal relationship between blood metabolites and the risk of two major CLDs, including primary biliary cholangitis (PBC) and primary sclerosing cholangitis (PSC).

**Methods:**

Univariable and multivariable Mendelian randomization (MR) approaches were employed to uncover potential causal associations between blood metabolites and 2 CLDs, including PBS and PSC, through extracting instrumental variables (IVs) for metabolites from genome-wide association studies (GWAS) conducted on European individuals. The GWAS summary data of PBC or PSC were sourced from two distinct datasets. The initial analysis employed inverse variance weighted (IVW) and an array of sensitivity analyses, followed by replication and meta-analysis utilizing FinnGen consortium data. Finally, a multivariable MR analysis was carried out to ascertain the independent effects of each metabolite. Furthermore, the web-based tool MetaboAnalyst 5.0 was used to perform metabolic pathway examination.

**Results:**

A genetic causality between 15 metabolites and CLDs was recognized after preliminary analysis and false discovery rate (FDR) correction. Subsequently, 9 metabolites consistently represented an association through replication and meta-analysis. Additionally, the independent causal effects of 7 metabolites were corroborated by multivariable MR analysis. Specifically, the metabolites isovalerylcarnitine (odds ratio [OR] = 3.146, 95% confidence intervals [CI]: 1.471–6.726, *p* = 0.003), valine (OR = 192.44, 95%CI: 4.949–7483.27, *p* = 0.005), and mannose (OR = 0.184, 95%CI: 0.068–0.499, *p* < 0.001) were found to have a causal relationship with the occurrence of PBC. Furthermore, erythrose (OR = 5.504, 95%CI: 1.801–16.821, *p* = 0.003), 1-stearoylglycerophosphocholine (OR = 6.753, 95%CI: 2.621–17.399, *p* = 7.64 × 10^−5^), X-11847 (OR = 0.478, 95%CI: 0.352–0.650, *p* = 2.28 × 10^−6^), and X-12405 (OR = 3.765, 95%CI: 1.771–8.005, *p* = 5.71 × 10^−4^) were independently associated with the occurrence of PSC. Furthermore, the analysis of metabolic pathways identified seven significant pathways in two CLDs.

**Conclusion:**

The findings of the present study have unveiled robust causal relationships between 7 metabolites and 2 CLDs, thereby providing novel insights into the metabolic mechanisms and therapeutic strategies for these disorders.

## Graphical Abstract

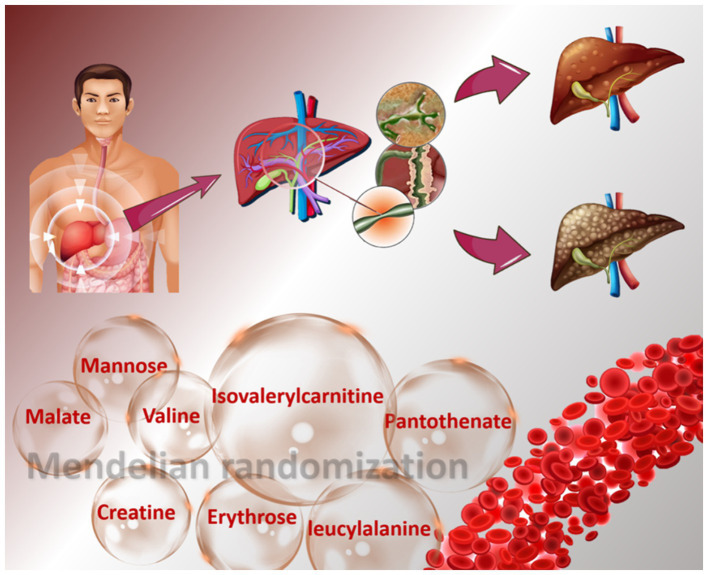

## 1 Introduction

Autoimmune cholestatic liver diseases (CLDs) are rare hepatic disorders characterized by progressive inflammatory destruction of the bile ducts. Primary biliary cholangitis and sclerosing cholangitis, known as PBC and PSC, are the most frequent CLDs, leading to a high rate of mortality and morbidity in patients with liver disorders. These conditions may progress to cirrhosis or hepatocellular carcinoma (1). Based on a report published in 2021, PBC and PSC cases are in the ranges from 1.91 to 40.2 and 0.78 to 31.7 patients per 100,000 individuals in the Europe, North America, and the Asia-Pacific regions, respectively (2). Despite their rarity, such diseases impose a disproportionately high clinical burden compared to their population-based incidence and prevalence rates. Accordingly, early diagnosis and therapy are required to lessen the stratification risk and promote follow-up treatment procedures. So far, however, there has been little discussion about the PBC and PSC etiology, mainly resulting from the complex interplay between environmental triggers and genetic susceptibility factors (3). The clinical progression of the mentioned conditions is influenced by several variables, making it challenging to design clinical trials. Therefore, it is crucial to identify modifiable risk factors and potential clinical interventions, capable of being implemented in the early stages of the diseases.

PBC is mainly denoted as a chronic condition, occurring when T-cells damage the biliary epithelium, leading to liver fibrosis and cirrhosis. The cause of PBC is not fully understood, but there are indications of a genetic component due to various immune function-related gene loci and an increased risk among monozygotic twins. Environmental factors, chemical exposure, infections, aberrant immune responses, and molecular mimicry have also been declared as potential contributors to PBC development. Autoantibodies, which are serologic hallmarks of PBC, play a vital role in the diagnosis and prognosis of the condition. Meanwhile, PSC is an ailment caused by damaging the bile ducts, leading to scarring, narrowing, cholestasis, and progressive liver damage. Although the exact reason for PSC has not been examined yet, its progress might be affected by the contribution of several factors, such as genetic risk loci in human leukocyte antigens, bacterial infection, gut microbiome changes, and environmental exposures (4).

The PBC or PSC diagnosis requires a series of examinations, such as serum biochemistry, serological biomarkers, imaging, and in some cases, liver biopsy. In recent years, blood metabolites have been associated with autoimmune diseases due to their non-invasive, diverse nature (5). The advent of high-throughput sequencing technologies and the rise of metabolomics have facilitated the extensive investigation of metabolites as functional intermediates, shedding light on their biological significance, in tandem with their relationships with diseases. A growing body of evidence has suggested the association of numerous metabolic features with the risk of pancreatic inflammation, Alzheimer’s, and cardiovascular diseases (6–8). Nevertheless, the study of the genetic and causal relationships between blood metabolites and the risk of CLDs has remained constrained. Additionally, despite the differences between PBC and PSC autoimmune diseases, some patients exhibit overlapping features with autoimmune hepatitis (AIH), such as PBC-AIH or PSC-AIH overlap syndrome (OS).

Considering the significance of early and accurate PBC and PSC diagnosis, this study aims to evaluate the role of blood metabolites in the risk of PBC and PSC, benefitting from univariable and multivariable Mendelian randomization (MR) analysis methods. The MR technique has emerged as a powerful and robust approach toward the investigation of potential causal relationships, owing to its distinct advantages, such as limited susceptibility to reverse causation and confounding effects, as well as the proper capacity to simulate randomized controlled trials (9). Correspondingly, a genetic correlation scan is conducted to investigate the relationship between blood metabolites and two CLDs using large-scale genome-wide association study (GWAS) summary data for metabolites and PBC/PSC. Based on the obtained results, this study might provide novel insights into the genetic mechanisms underlying PBC and PSC.

## 2 Materials and methods

### 2.1 Study design

**Figure 1:**
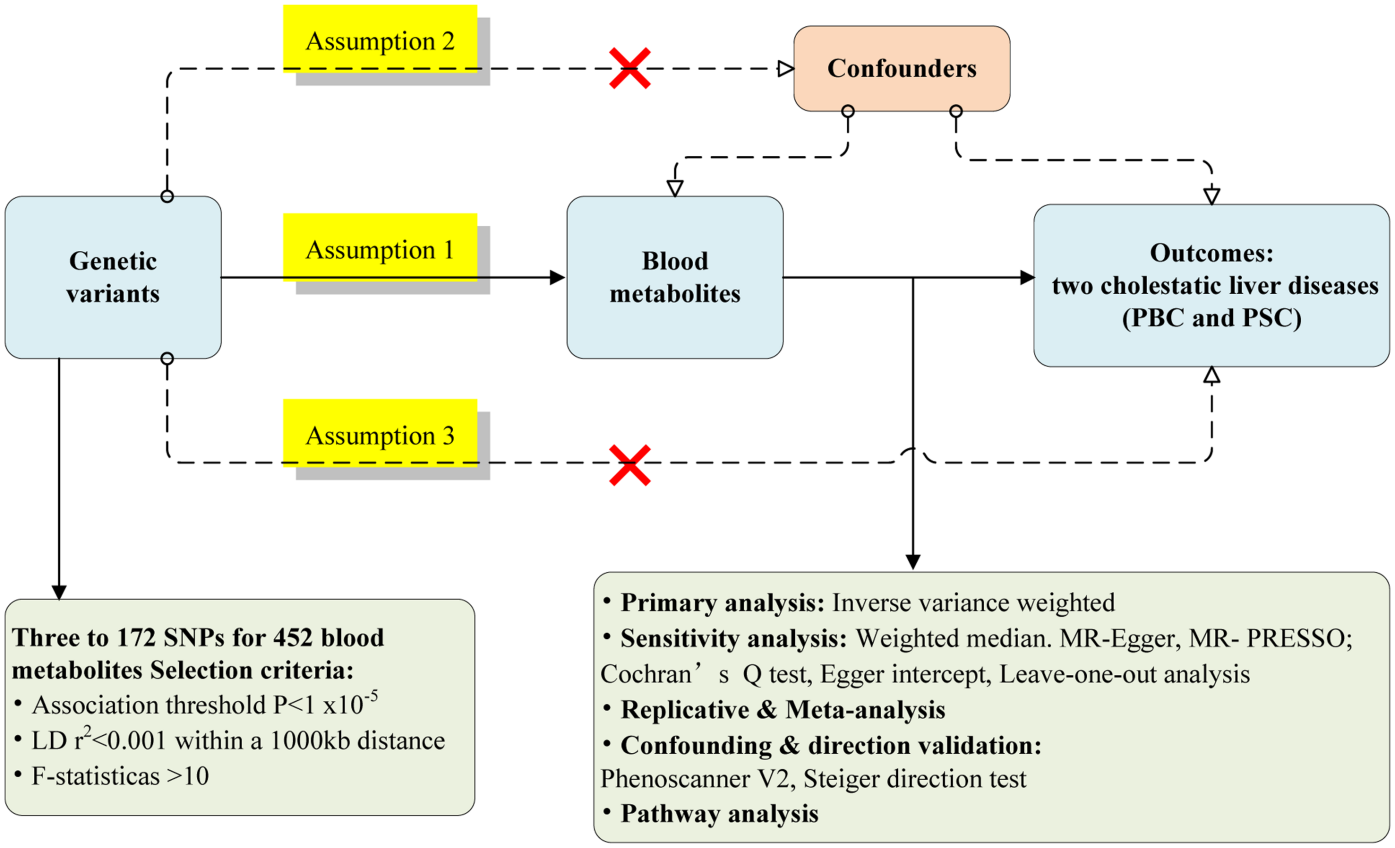
Flow chart for the Mendelian randomized analysis.

Figure 1 represents the flow chart of the Mendelian randomized analysis employed in this study. We postulated that 452 blood metabolites serve as exposures influencing susceptibility to CLDs. The GWAS data for the CLDs were obtained from two distinct GWAS consortia, showing the discovery cohort and replication sets, respectively.

Only metabolites with a significant causal relationship in the discovery cohort were included in the replication set for further analysis. A meta-analysis also combined the results from both sets. Finally, a multivariate MR analysis was carried out on the metabolites, representing causality in the meta-analysis, as well as identifying metabolites with independent causal associations with the two CLDs.

### 2.2 Summary statistics for metabolites and cholestatic liver diseases

The exposure data for metabolites comes from a 2014 metabolomic genome-wide association study by Shin et al. (10), which involved 7,824 European adult participants from TwinsUK and KORA cohorts. According to rigorous quality control procedures, more than 2.1 million SNPs and 452 metabolites were detected, comprising 275 and 177 individuals with known and unknown metabolites. The aggregated summary statistics for these metabolite GWAS results are publicly accessible with the ID of “met-a” on the MRBase website^1^.

The outcome dataset was collected from two separate GWAS cohorts, including the GWAS Catalog and the FinnGen consortium. The discovery set data for PBC came from the European GWAS, which included 2,764 cases and 10,475 controls (11). Additionally, the PSC data was obtained from a GWAS by the IPSCSG, involving 4,796 European cases and 19,955 population controls. More details can be found in the original GWAS study (12).

Summary statistics for the replication dataset were exclusively drawn from the FinnGen Release 8 database (13). This database is part of the FinnGen consortium, which gathers and analyzes genetic and health data from around half a million participants in the Finnish Biobank. The data from the FinnGen Alliance can be accessed publicly at https://r8.finngen.fi/pheno/. Data from the FinnGen Alliance is publicly accessible on the (https://r8.finngen.fi/pheno/, accessed on March 8, 2023) website. Table 1 summarizes specific details regarding the employed datasets.

**Table 1:** Source of outcome GWAS summary data.

Trait	Source	PMID	Cases	Control	GWAS ID/Phenocode	Population
Metabolites	IEU open GWAS project	24,816,252	7,824	NA	met-a-(303 to 754)	European
PBC	GWAS Catalog	26,394,269	2,764	10,475	GCST003129	European
	FinnGen Mrach 2023 Release		497	257,081	CHIRBIL_PRIM_R8	European
PSC	IPSCSG	27,992,413	4,796	19,955	GCST004030	European
	FinnGen Mrach 2023 Release		1,491	301,383	K11_CHOLANGI	European

GWAS, genome-wide association study; PBC, primary biliary cholangitis; PSC, primary sclerosing cholangitis; IPSCSG, International PSC Study Group.

### 2.3 Mendelian randomization analysis

#### 2.3.1 Primary MR analysis

In the first step, the valid single nucleotide polymorphisms (SNPs) were used as IVs, considering the limited number of genome-wide significant SNPs. A relaxed threshold of *p* < 1 × 10^−5^ was employed to identify the IV target, a commonly utilized method in prior MR studies (14, 15). Subsequently, the clumping function was applied to perform linkage disequilibrium screening on the selected IVs, using a threshold of 10,000 KB and *R*^2^ < 0.001. Additionally, the *F*-statistic is a statistical measure that quantifies both the magnitude and accuracy of the genetic impact on the trait. It can be calculated as *F* = *R*^2^ (*N* − 2)/(1 − *R*^2^), where *R*^2^ represents the proportion of variance in the trait explained by the IVs, and *N* denotes the sample size of GWAS involving SNPs with the trait (16). SNPs were filtered with *F*-statistics exceeding 10 for further analysis, as it can significantly mitigate the risk of IV bias.

In the second phase, SNPs were extracted from the consistent data with the exposure. Proxy variants with an *R*^2^ > 80% were sought in the European reference ancestral population from the 1,000 Genomes Project in the SNPs’ absence cases in the outcome (17). Simultaneously, the following two SNP types, including missing IVs in the outcome and lacking corresponding proxy SNPs, as well as palindromic SNPs were discarded. All available IVs are shown in Supplementary Table S1. In the MR analysis, the random-effects model was employed within the inverse variance weighted (IVW) method as the primary approach to estimate the blood metabolite impacts on PBC and PSC. This method operates under the assumption that all genetic variations serve as valid instruments (18), allowing for robust causal estimations without being influenced by directional pleiotropy. Additionally, the false discovery rate (FDR) correction was applied to ensure the reliability of our findings. If the adjusted *p*-value was less than 0.05 after the Benjamini–Hochberg correction for the causal effect of a specific metabolite, it was considered to have a statistically significant association. Conversely, an original *p*-value <0.05 was interpreted with a corrected *p*-value >0.05 as a potential correlation.

In the third section, a series of sensitivity examinations were conducted on the initially identified metabolites of interest to ensure the robustness of the findings. Among them, weighted median (WM) (19) and MR-Egger (20) were utilized as Supplementary methods. Metabolites displaying consistent directional effects across IVW, MR-Egger, and WM techniques were further investigated in sensitivity evaluation. Cochran’s *Q* test (21) and Egger intercept (20) were also employed to figure out heterogeneity and horizontal pleiotropy, respectively. In the presence of pleiotropy, the MR-PRESSO test was applied to identify and exclude outliers (22), followed by a subsequent MR analysis round. The MR Steiger test was performed to assess the direction of observed causal relationships for the metabolites of interest and eliminate those potentially indicative of reverse causality (23). These sensitivity analyses refined the list of candidate metabolites, minimizing potential issues.

#### 2.3.2 Confounding assessments

The phenoscannerV2 website^2^ was employed to enhance the MR hypothesis. The main goal was to examine the probable association of the IVs for the metabolites with potential confounding factors, like BMI (24), cholesterol, and triglycerides (25). Following this, potentially confounded SNPs were excluded and the IVW analysis was re-executed.

#### 2.3.3 Replication and meta-analysis

Replication investigations were carried out using two additional independent GWAS datasets for PBC and PSC from the FinnGen consortium to affirm the reliability of our candidate metabolites. Correspondingly, meta-analysis was employed to combine the outcomes from these datasets and identify the final candidates.

#### 2.3.4 Multivariate and reverse MR analysis

The study also employed multivariate MR analysis to investigate the effect of multiple metabolites on the same outcome. Then, reverse IVW analysis was performed for those candidate metabolites identified as independently affecting the outcome in the multivariate MR analysis. This involved considering PBC or PSC as exposures and the metabolites as outcomes to explore whether there is a reverse causality to determine the disease’s impact on metabolites. The IVs for PBC and PSC underwent a selection process similar to the prescribed one, with a genome-wide significance threshold set at 5 × 10^−8^.

#### 2.3.5 Metabolic pathway analysis

Finally, the MetaboAnalyst 5.0^3^ online tool was used to perform an extensive metabolic pathway analysis of the identified metabolites with potential associations to the two CLDs. We used the Small Molecule Pathway Database (SMPDB) containing 99 metabolite sets, in tandem with 84 metabolite groups from the Kyoto Encyclopedia of Genes and Genomes (KEGG) database, to find potential metabolic pathways for PBC or PSC biological processes.

### 2.4 Statistical analysis

Statistical analysis was performed using R software (version 4.2.3). Regarding univariable MR analysis, the “TwoSampleMR” package was employed, while the “Mendelian Randomization” and “MVMR” packages were utilized in R for multivariable MR analysis. METAL (23) (version 2011-03-25) was used to examine the meta-analyses of the obtained outcomes. Additionally, FDR correction was applied to mitigate the risk of false positives in multiple tests. A causal effect of a specific metabolite was deemed statistically significant when the associated FDR estimate was less than 0.05.

## 3 Results

### 3.1 Primary MR analyses

**Figure 2:**
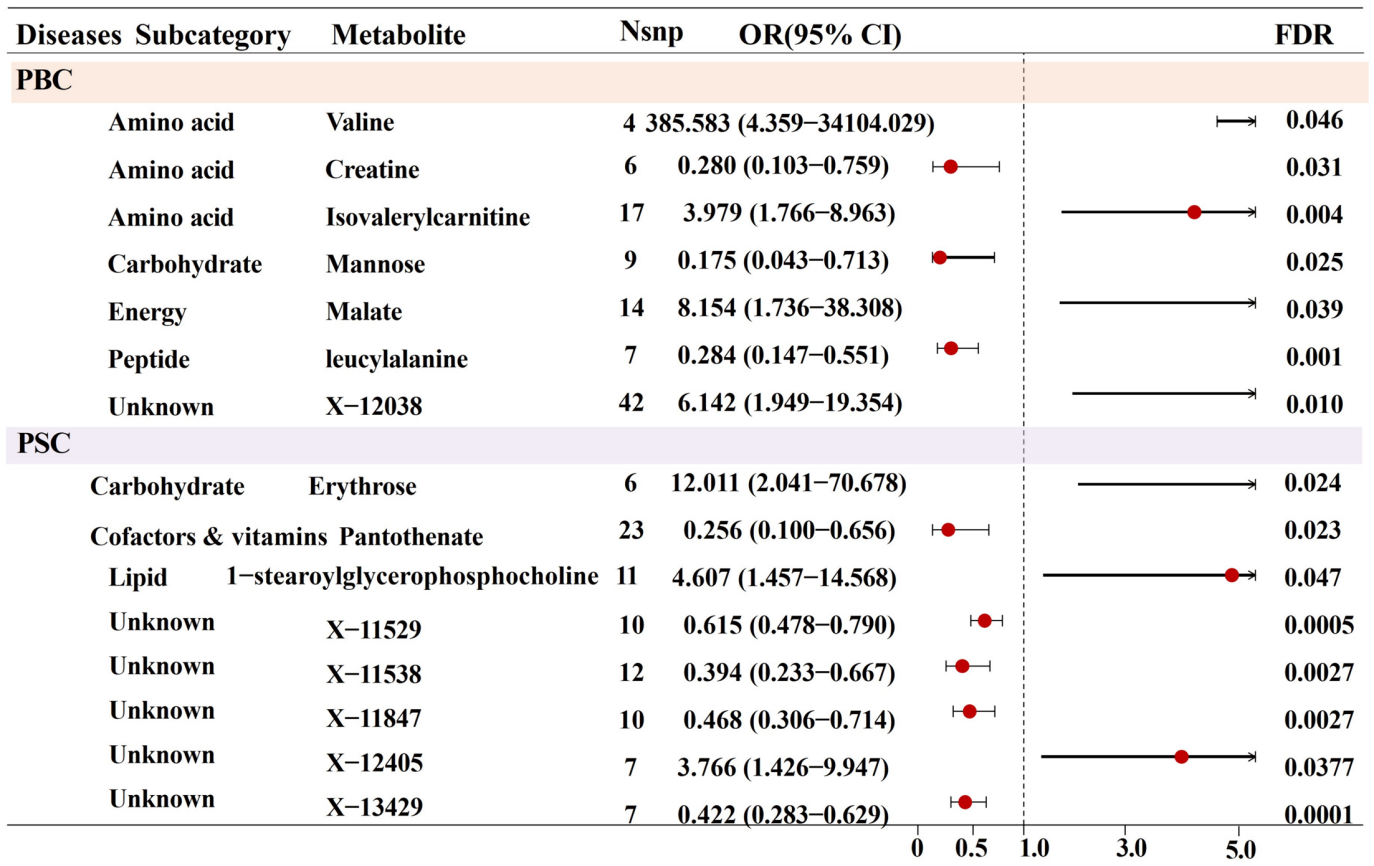
Forest plot for the causal effect of identified metabolites on the risk of PBC and PSC cholestatic liver diseases derived from inverse variance weighted (IVW).

After completing the initial tool variable selection, several metabolite IV counts were observed, ranging from 3 to 172 with a median of 15. Subsequently, the selected IVs were used to conduct an initial univariate MR analysis toward exploring potential causal relationships between the metabolites and PBC and PSC. Using the MR-IVW analysis method, 54 potential associations were preliminarily identified, involving 53 different metabolites (*p* < 0.05) (see Supplementary Table S2). Specifically, metabolites associated with PBC included fifteen known and nine unknown metabolites, respectively. Meanwhile, the ones associated with PSC contained eighteen known metabolites and twelve unknown metabolites, along with one metabolite named erythrose, representing potential associations with both PBC and PSC. It is worth noting that only six and one known and unknown metabolites were, respectively, associated with PBC (FDR < 0.05) after several testing corrections. Additionally, PSC was significantly linked with three known and five unknown metabolites (FDR < 0.05), as is displayed in Figure 2. It is essential to emphasize that no metabolites were found to be significantly related with both of the 2 CLDs, after multiple corrections. To be specific, seven metabolite cases have been recognized to establish causal links with PBC, including valine (odds ratio [OR] = 385.583, 95% confidence intervals [CI]: 4.359–34104.030, FDR = 0.046), creatine (OR = 0.280, 95%CI: 0.103–0.759, FDR = 0.031), isovalerylcarnitine (IVC) (OR = 3.979, 95%CI: 1.766–8.963, FDR = 0.004), mannose (OR = 0.175, 95%CI: 0.043–0.713, FDR = 0.025), malate (OR = 8.153, 95%CI: 1.736–38.308, FDR = 0.039), leucylalanine (OR = 0.284, 95%CI: 0.147–0.551, FDR < 0.001), and X-12038 (OR = 6.142, 95%CI: 1.949–19.354, FDR = 0.010). Moreover, casual associations were examined between eight metabolites and PSC, containing erythrose (OR = 12.010, 95%CI: 2.041–70.678, FDR = 0.024), pantothenate (OR = 0.256, 95%CI: 0.100–0.656, FDR = 0.023), 1-stearoylglycerophosphocholine (OR = 4.607, 95%CI: 1.457–14.568, FDR = 0.047), X-11529 (OR = 0.615, 95%CI: 0.478–0.790, FDR < 0.001), X-11538 (OR = 0.394, 95%CI: 0.233–0.667, FDR = 0.003), X-11847 (OR = 0.468, 95%CI: 0.306–0.714, FDR = 0.002), X-12405 (OR = 3.765, 95%CI: 1.426–9.947, FDR = 0.037), and X-13429 (OR = 0.422, 95%CI: 0.283–0.629, FDR < 0.001).

For the preliminary screening of candidate metabolites, a series of sensitivity examinations was carried out to enhance the robustness of the MR primary investigation. As is observable in Supplementary Table S3 and Supplementary Figure S1, nine known and six unknown metabolites exhibited steady direction and amplitude in IVW, MR-Egger, and weighted median analysis, corroborating the consistency of assessments across various testing methods for the mentioned metabolites. Subsequently, the results were subjected to pleiotropy and heterogeneity scrutiny, where all Cochran’s *Q* heterogeneity test *p*-values were greater than 0.05, indicating the absence of significant heterogeneity. Moreover, the small intercepts and *p*-values in MR-Egger suggested the negligible impact of horizontal pleiotropy. Also, the *p*-values obtained from the MR PRESSO-outlier test were greater than 0.05, supporting a very low probability of horizontal pleiotropy and the presence of outliers (Supplementary Table S3). The attained result tied well with the leave-one-out analysis presented in Supplementary Figure S2. Furthermore, Steiger directional tests were performed to validate the direction of influence from metabolites to the considered CLDs, exhibiting extremely minimal likelihood of statistical bias from reverse causation (Table 2).

**Table 2:** Sensitivity analysis for the causal association between blood metabolites and cholestatic liver diseases.

Metabolites	Diseases	Subcategory	Heterogeneity test		Pleiotropy test			FDR_IVW_	Steiger test	
			IVW		MR-Egger				Correct causal direction	Steiger_pval
			Q (*I*^2^)	Q_pval	Intercept	SE	*p*			
Mannose	PBC	Carbohydrate	12.871 (37.85%)	0.116	−0.012	0.035	0.743	0.025	TRUE	3.0309 × 10^−73^
Malate	PBC	Energy	15.514 (16.20%)	0.276	0.015	0.060	0.813	0.039	TRUE	3.37241 × 10^−36^
Valine	PBC	Amino acid	0.500 (0.00%)	0.919	−0.013	0.037	0.758	0.046	TRUE	2.61682 × 10^−21^
Creatine	PBC	Amino acid	3.620 (0.00%)	0.605	0.024	0.036	0.544	0.031	TRUE	1.2098 × 10^−34^
X-12038	PBC	Unknown	41.465 (1.12%)	0.450	0.010	0.032	0.742	0.010	TRUE	1.13 × 10^−115^
Isovalerylcarnitine	PBC	Amino acid	8.442 (0.00%)	0.935	0.012	0.022	0.600	0.004	TRUE	9.53331 × 10^−96^
leucylalanine	PBC	Peptide	4.567 (0.00%)	0.600	−0.057	0.064	0.414	0.001	TRUE	2.27609 × 10^−34^
Pantothenate	PSC	Cofactors and vitamins	14.693 (0.00%)	0.875	−0.007	0.018	0.700	0.023	TRUE	1.03623 × 10^−98^
Erythrose	PSC	Carbohydrate	7.650 (34.64%)	0.177	−0.056	0.107	0.628	0.024	TRUE	1.39181 × 10^−11^
X-11529	PSC	Unknown	8.406 (0.00%)	0.494	0.020	0.017	0.276	0.000	TRUE	6.6887 × 10^−176^
X-11538	PSC	Unknown	4.533 (0.00%)	0.952	0.017	0.022	0.465	0.003	TRUE	1.0065 × 10^−100^
X-11847	PSC	Unknown	3.489 (0.00%)	0.942	−0.018	0.033	0.610	0.002	TRUE	6.79813 × 10^−25^
X-12405	PSC	Unknown	3.219 (0.00%)	0.781	−0.014	0.050	0.797	0.037	TRUE	4.1679 × 10^−33^
1-stearoyl glycerophosphocholine	PSC	Lipid	3.792 (0.00%)	0.956	−0.005	0.029	0.858	0.047	TRUE	6.28367 × 10^−44^
X-13429	PSC	Unknown	4.824 (0.00%)	0.567	0.020	0.038	0.628	0.000	TRUE	3.50935 × 10^−73^

IVW, inverse variance weighted; MR, Mendelian randomization; FDR, false discovery rate; PBC, primary biliary cholangitis; PSC, primary sclerosing cholangitis.

### 3.2 Confounding assessment

Although a series of sensitivity examinations revealed no biases with ineffective MR estimations, potential confounding factors related to the metabolite instrument variables, such as BMI, cholesterol, and triglycerides, were proactively investigated. Through a search on the Phenoscanner, one SNP (rs1260326) was identified to associate with triglycerides for the mannose metabolite. After excluding this SNP, the MR-IVW analysis was conducted again, and the results continued to demonstrate a significant causal relationship between mannose and the PBC risk (IVW OR = 0.739, 95% CI: 0.547–0.994, FDR = 0.045).

### 3.3 Replication and meta-analysis

**Figure 3:**
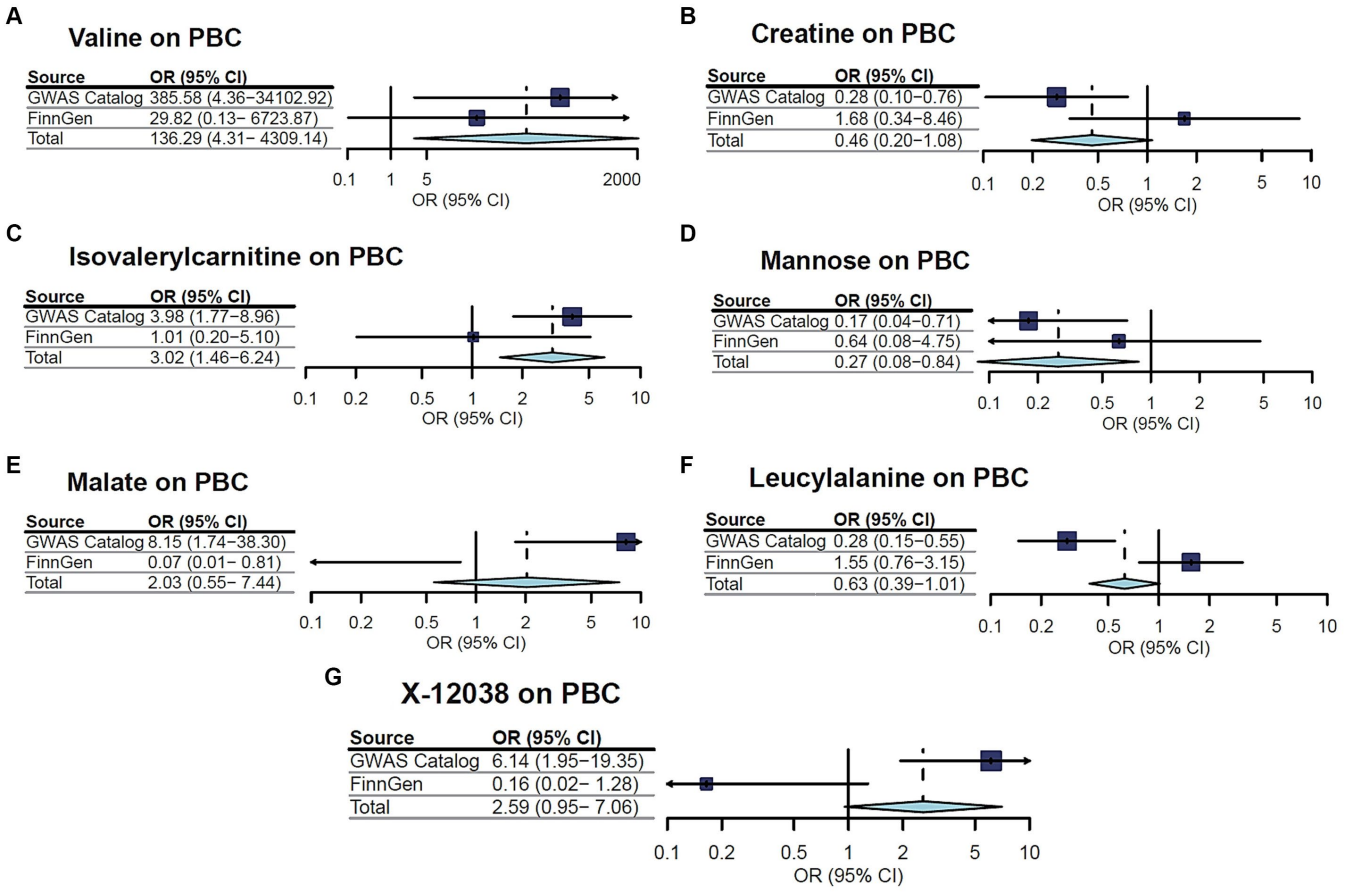
Meta-analysis of the causal associations between metabolites and primary biliary cholangitis (PBC).

**Figure 4:**
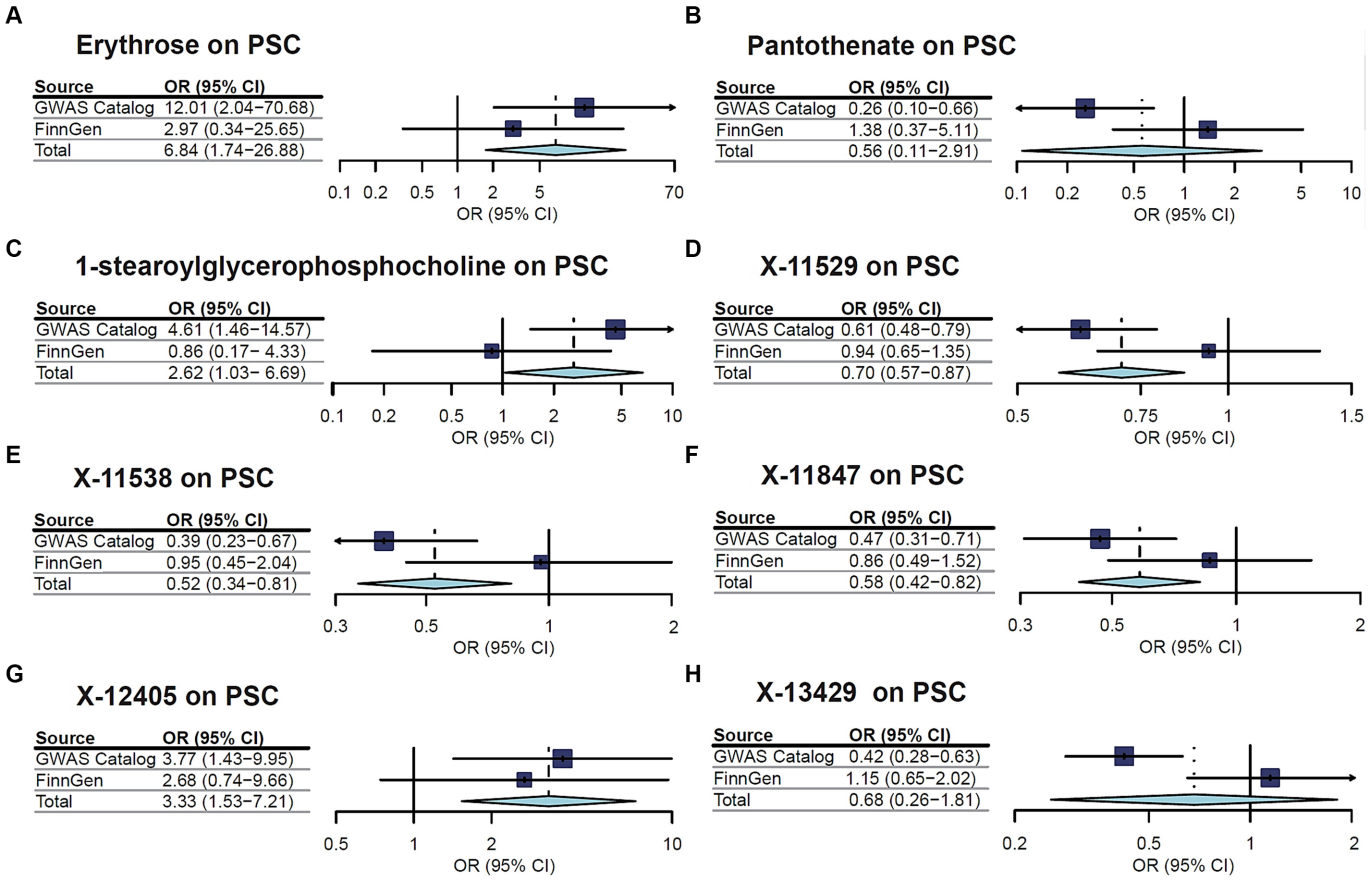
Meta-analysis of the causal associations between metabolites and primary sclerosing cholangitis (PSC).

A replication analysis using two comparable GWAS summary datasets sourced from the FinnGen consortium was embarked on for further substantiation of the causal link between the identified metabolites and the risk of two CLDs, including PBC and PSC. The results unveiled a consistent pattern in the relationship between certain metabolites and the susceptibility to PBC and PSC. Specifically, out of the seven metabolites initially associated with PBC, three cases demonstrated a congruent trend in the replication analysis, encompassing valine, isovalerylcarnitine, and mannose. Additionally, among the eight metabolites linked to PSC, five showcased trends in concordance with the initial findings. As is depicted in Figures 3, 4, a meta-analysis of the findings from these two datasets further solidified several outcomes. First, valine (OR = 136.2889, 95%CI: 4.310–4309.143, *p* = 0.005) and isovalerylcarnitine (OR = 3.019, 95%CI: 1.461–6.237, *p* = 0.003) were causally associated with an increased risk of PBC at the genetic level, whereas mannose (OR = 0.267, 95%CI: 0.084–0.844, *p* = 0.02) was causally linked with a decreased risk of PBC. In addition, erythrose (OR = 6.837, 95%CI: 1.739–26.882, *p* = 0.006), 1-stearoylglycerophosphocholine (OR = 2.620, 95%CI: 1.026–6.691, *p* = 0.04), and X-12405 (OR = 3.326, 95%CI: 1.534–7.214, *p* = 0.002) were identified as PSC risk factors. Moreover, three undisclosed metabolites, specifically X-11529 (OR = 0.704, 95%CI: 0.573–0.866, *p* < 0.001), X-11538 (OR = 0.525, 95%CI: 0.341–0.809, *p* = 0.003), and X-11847 (OR = 0.583, 95%CI: 0.415–0.819, *p* = 0.002) exhibited a protective effect against PSC development.

### 3.4 Multivariate and reverse MR analysis

The results of our meta-analysis have illuminated causal relationships between several metabolites and PBC or PSC, thereby, a multivariate MR analysis was employed to investigate the independent effects of each metabolite. Accordingly, seven out of nine metabolites exhibited independent impacts. It is worth noting that the multivariate MR of the three linked metabolites to PBC aligns harmoniously in both direction and magnitude with the unadjusted outcomes obtained from univariate MR analysis (see Table 3). Remarkably, three metabolites represented independent causal effects on PBC susceptibility, including isovalerylcarnitine (OR = 3.146, 95%CI: 1.471–6.726, *p* = 0.003), valine (OR = 192.44, 95%CI: 4.949–7483.27, *p* = 0.005), and mannose (OR = 0.184, 95%CI: 0.068–0.499, *p* < 0.001). Among six associated metabolites with PSC, the causal influence of two unidentified metabolites (X-11529 and X-11538) showed a weakening effect on PSC after multivariate MR analysis. Meanwhile, multivariate MR analysis further substantiated the independent causal effects of the other four metabolites on PSC, containing erythrose (OR = 5.504, 95%CI: 1.801–16.821, *p* = 0.003), 1-stearoylglycerophosphocholine (OR = 6.753, 95%CI: 2.621–17.399, *p* = 7.64 × 10^−5^), X-11847 (OR = 0.478, 95%CI: 0.352–0.650, *p* = 2.28 × 10^−6^), and X-12405 (OR = 3.765, 95%CI: 1.771–8.005, *p* = 5.71 × 10^−4^).

**Table 3:** Estimated causal effects of metabolites on cholestatic liver diseases by the multivariable MR analysis.

Metabolite	Diseases	Nsnp	Multivariable MR	
			OR (95% CI)	*p*
Mannose	PBC	32	0.184 (0.068–0.499)	8.807 × 10^−4^
Valine	PBC	32	192.442 (4.949–7483.270)	4.859 × 10^−3^
Isovalerylcarnitine	PBC	32	3.146 (1.471–6.726)	3.114 × 10^−3^
Erythrose	PSC	54	5.504 (1.801–16.821)	2.772 × 10^−3^
X-11529	PSC	54	0.812 (0.572–1.153)	2.442 × 10^−1^
X-11538	PSC	54	0.582 (0.267–1.269)	1.738 × 10^−1^
X-11847	PSC	54	0.478 (0.352–0.650)	2.280 × 10^−6^
X-12405	PSC	54	3.765 (1.771–8.005)	5.715 × 10^−4^
1-stearoyl glycerophosphocholine	PSC	54	6.753 (2.621–17.399)	7.640 × 10^−5^

MR, Mendelian randomization; OR, odds ratio; CI, confidence interval; PBC, primary biliary cholangitis; PSC, primary sclerosing cholangitis.

Subsequently, additional reverse MR investigations were embarked on using instrumental variables for PBC and PSC. In the considered reversed causal investigation, PBC and PSC played the role of exposures, while the seven metabolites with independent impacts served as outcomes. Independent SNPs were selected as instrumental variables with a significance level of *p* < 5 × 10^−8^ and conducted IVW-MR estimation, as is detailed in Supplementary Table S4. Based on the findings, a scarcity of substantial evidence supporting reverse causal relationships is suggested between these metabolites and either PSC or PBC, aligning with the earlier Steiger results.

Furthermore, we conducted additional analysis to examine the causal relationships between the 7 metabolites and the outcomes at a stricter threshold (*p* < 5 × 10^−6^) (see Supplementary Table S5). Our analysis under stricter threshold conditions using the IVW method reveals that the results for six metabolites are consistent with the initial analysis direction, with four of these showing a significant causal effect on the outcomes (*p* < 0.05). However, the metabolite Erythrose, which is supported by only one valid SNP, demonstrates a disappearance of the causal relationship with PSC. This may be attributed to bias arising from the insufficient number of SNPs.

### 3.5 Metabolic pathway analysis

To provide a clearer and more comprehensive understanding of all the enriched metabolic pathways, we have compiled all the enriched pathways into Supplementary Table S6. Table 4 summarizes the results significantly enriched (*p* < 0.05) after metabolic pathway analysis, highlighting seven important pathways found in two CLDs. According to the findings, “Valine, leucine, and isoleucine biosynthesis” (*p* = 0.021), “Malate–Aspartate Shuttle” (*p* = 0.034), and “Pantothenate and CoA biosynthesis” (*p* = 0.048) pathways were linked to the PBC pathogenesis. Simultaneously, “Valine, leucine, and isoleucine biosynthesis” (*p* = 0.015), “Linoleic acid metabolism” (*p* = 0.016), “Arginine biosynthesis” (*p* = 0.027), and “Bile Acid Biosynthesis” (*p* = 0.043) pathways were considered relevant to PSC. It is intriguing to note that PBC and PSC share a common metabolic pathway, namely, “Valine, leucine, and isoleucine biosynthesis.” Correspondingly, the obtained discoveries provided further insights into the metabolic mechanisms underlying CLDs.

**Table 4:** Significant metabolic pathways involved in the 2 cholestatic liver diseases.

Traits	Metabolites pathway	Involved metabolites	Total	*p* value	Database
PBC	Valine, leucine, and isoleucine biosynthesis	Valine	8	0.021	KEGG
PBC	Malate-Aspartate Shuttle	Malate	7	0.034	SMPDB
PBC	Pantothenate and CoA biosynthesis	Valine	19	0.048	KEGG
PSC	Valine, leucine, and isoleucine biosynthesis	Leucine	8	0.015	KEGG
PSC	Linoleic acid metabolism	Linoleate (18:2n6)	5	0.016	KEGG
PSC	Arginine biosynthesis	Urea	14	0.027	KEGG
PSC	Bile Acid Biosynthesis	Palmitate (16:0), Deoxycholate	59	0.043	SMPDB

PBC, primary biliary cholangitis; PSC, primary sclerosing cholangitis; KEGG, Kyoto encyclopedia of genes and genomes; SMPDB, small molecule pathway database.

## 4 Discussion

The current MR study delves into the promising associations between two CLDs and metabolites. Based on the conducted analysis, seven metabolites revealed statistical significance even after multiple testing corrections, encompassing two previously unidentified compounds. Additionally, reverse causation exclusion was carried out, affirming that the severe candidate metabolites were indeed causative factors for the two CLDs, rather than outcomes. Also, seven significant metabolic pathways were associated with the PBC and PSC, including one shared pathway. Accordingly, the study represented a comprehensive and systematic evaluation of the causal effects of blood metabolites and metabolic pathways on CLDs using MR analysis. In addition, the reliability and consistency of the findings were ensured by conducting replication analysis across various databases. Therefore, fresh insights were offered into the role of gene–environment interactions in the pathogenesis of CLDs, providing potential inspiration for further mechanistic exploration.

Based upon the results, mannose was the initial metabolite playing as a protective factor against the occurrence of PBC. Few previous studies have focused on the correlation of PBC with blood metabolite mannose levels. In 2021, Franssen et al. demonstrated that mannose effectively prevents urinary tract infections via a randomized controlled trial (26). Gonzalez et al. also declared mannose’s ability to inhibit tumor growth by suppressing glucose metabolism using *in vitro* examinations (27). As is shown in the Zhang et al. study, mannose also exhibits a positive impact in immunotherapy (28) and influences the glycosylation of PD-L1, promoting its degradation and thereby enhancing the efficacy of immunotherapy in Triple-negative breast cancer. These findings align well with the obtained results in the current investigation, providing indirect support for mannose’s potential protective role in AIDs. At a genetic level, elevated mannose levels may contribute to reducing the PBC risk. Furthermore, another *in vitro* experiment has identified a possible mechanism, wherein mannose activates TGF-β to increase the production of regulatory T cells (Treg), subsequently suppressing AIDs (29). This suggests that mannose may maintain immune system balance by promoting PD-L1 degradation, regulating Treg expansion, and diminishing attacks on self-tissues, ultimately reducing the risk of PBC development. Nevertheless, the specific mechanisms and signaling pathways bridging mannose to PBC onset require further in-depth investigation. This discovery offers valuable leads for future research and treatments.

Another remarkable finding gained in this evaluation was the association of two genetic-level risk factors with PBC, IVC, and valine. IVC is a carnitine substrate for isovaleryl-CoA dehydrogenase, which represents a specific acylcarnitine involved in early-stage cellular immunity, enhancing phagocytosis and cytotoxicity (30). Acylcarnitines are involved in fatty acid β-oxidation and the branched-chain amino acid metabolism, including valine, leucine, and isoleucine, associated with the progression of AIDs (31, 32). Observational research has established a positive correlation between blood acylcarnitine levels and peripheral neuropathy in type 2 diabetes patients (33). It is also suggested that IVC may accelerate the progression of rheumatoid arthritis (4). Another study by Jewell et al. also offered a potential explanation for this phenomenon, as IVC is involved in the degradation of leucine and fatty acids. Leucine is an essential amino acid that regulates cell proliferation and metabolism through the mTORC1 complex, thereby influencing disease development (34). PBC is characterized primarily by autoimmune factors, leading to hepatocyte and/or bile duct injury, in tandem with disruptions to metabolic balance within the body (35). Based on the findings attained from the current evaluation, IVC exerts a promotional effect on PBC occurrence at the genetic level as a circulating blood metabolite. This effect may be associated with IVC’s involvement in cellular immunity, enhancing phagocytosis and cytotoxicity.

As an essential amino acid, valine has been the subject of observational studies. Meanwhile, few research cases have explored the correlation between valine and the onset and PBC progression. In a study carried out in 2019, White et al. indicated a positive association between elevated levels of branched-chain amino acids in the blood with insulin resistance, as well as diabetes (5). Gu et al. also found that blood valine serves as an independent risk factor for non-alcoholic fatty liver disease (36). Accordingly, valine might have adverse implications for disease development, which is consistent with the findings obtained from the MR evaluation. Therefore, it is highly recommended that valine could be a potential therapeutic target, providing valuable leads toward deploying novel approaches for PBC treatment after further clinical examinations. It is crucial to emphasize that ursodeoxycholic acid (UDCA) did not exhibit a genetic-level association with the disease in the metabolomic screening, while it has been widely recognized as a frontline treatment for PBC. This discrepancy might be attributed to the fact that UDCA primarily addresses the bile stasis-induced hepatic injury critical to disease progression, rather than being linked to the genetic determinants of disease risk.

As another outcome of the present investigation, three genetic factors associated with PSC were recognized, including erythrose, 1-stearoylglycerophosphocholine, and an unidentified metabolite, X-12405. According to the literature, the correlation between erythrose and PSC is currently incomplete. Nonetheless, some studies have indicated that aldose metabolism facilitates the transition from liver fibrosis to liver cancer (37), and erythrose falls under the category of aldoses, implying its potential involvement in PSC onset. 1-stearoylglycerophosphocholine is a subtype of lysophosphatidylcholine (LPC), which is predominantly secreted by the liver (38). LPC levels can increase during inflammatory conditions and exhibit diverse effects depending on the environment. Conflicting results have been reported yet by the observational studies, in which some cases have recommended the ability of LPC upregulation to modulate immune checkpoint regulation, linking to enhanced survival rates in patients with acute liver failure (39). Conversely, other studies have proposed a contrary perspective, indicating that LPC exacerbates allergic reactions through promoting neutrophil infiltration and inclining IL17 expression (40), which implies its pro-inflammatory role. Based on the MR study findings, genetically elevated levels of 1-stearoylglycerophosphocholine promote PSC development, which aligns with its role in promoting inflammation and provides a new direction for early PSC prevention and treatment.

Moreover, the performed MR Analysis corroborated a causal relationship between two unknown metabolites and the PSC occurrence. Specifically, X-12405 was found to be linked with a higher risk of PSC, while X-11847 played a protective role in PSC development. However, it was challenging to extract further significant insights, resulting from a lack of information regarding the structure and function of these metabolites. It is worth noting that the successful identification of these unknown metabolites will greatly advance the discovery of biomarkers and research into cholestatic liver diseases.

According to the results gained through the present investigation, seven causal metabolic pathways were identified associated with the development of two CLDs. Interestingly, it was discovered that PBC and PSC share one crucial metabolic pathway, namely, valine, leucine, and isoleucine biosynthesis, playing pivotal roles in the disease progression. In an animal model study by Bandt et al., a close association was revealed between valine, leucine, and isoleucine biosynthesis with abnormalities in glucose and lipid metabolism, potentially inducing insulin resistance (41). The induction of autophagy by reducing TORC1 activity could be a possible mechanism for this occurrence (42). Furthermore, the enrichment of the pantothenate and CoA biosynthesis pathway has been linked significantly to colorectal cancer (43). Considering the essential roles, the observed imbalances in these pathways, regarding both PBC and PSC, suggest a significant disruption in material and energy metabolism. Regulating these metabolic pathways may offer potential strategies for preventing or treating the development of CLDs, such as PBC and PSC.

Overall, several bright sides were observed through the present evaluation. First, seven out of 452 metabolites were identified, exhibiting a causal relationship with the risk of developing either PBC or PSC. The considered MR analysis employed a diverse range of analytical methods to eliminate the potential for reverse causality, as well as confounding factors. Second, a meta-analysis of results was conducted from various databases, mitigating disparities stemming from distinct data sources, thus bolstering the credibility of our inferences regarding the causal links between metabolites and PBC or PSC. Third, potential therapeutic targets for cholestatic liver diseases were represented. The identified metabolites and pathways could serve as valuable circulating metabolic biomarkers for clinical screening and prevention of PBC and PSC. Fourth, the attained findings might contribute to enhancing healthcare professionals’ awareness of the significance of diet and lifestyle in liver disease prevention. Increasing understanding regarding the roles of specific metabolites, such as valine and mannose, in disease development can encourage high-risk individuals to opt for healthier dietary choices and lifestyle modifications.

It is worth noting that the present study has faced some limitations, recommended to be addressed in future investigations. First, it predominantly relied on data from random populations, which may potentially hinder the generalizability of the study findings. Future research is recommended to contemplate the inclusion of more diverse population samples to enhance the external validity of the results. Second, the reliance on a limited number of SNPs reaching genome-wide significance might introduce bias into the analysis. To address this concern, the threshold was declined, and the F statistic for each SNP exceeded 10. Additionally, the true direction derived from the Steiger test also supported the validity of SNPS with more relaxed *p*-values. Third, while MR methodology excels in causal inference, it is essential to remember that the findings from this MR study should undergo further validation through randomized controlled trials involving larger and more diverse populations.

## 5 Conclusion

In summary, the present study conducted a preliminary analysis and FDR correction to identify a genetic causality between 15 metabolites and two CLDs, including PBS and PSC. Subsequently, nine metabolites were identified with consistent association through replication and meta-analysis. Among the recognized metabolites, mannose emerged as a protective factor against the PBS occurrence. Mannose exhibited the potential to regulate Treg expansion, promote PD-L1 degradation, and reduce attacks on self-tissues, ultimately reducing the risk of PBC development. Additionally, two genetic-level risk factors were detected in the association with PBC. The circulating blood metabolite IVC exerted a promotional effect on the occurrence of PBC at the genetic level, which may be linked with cellular immunity. Besides, this study employed bidirectional and multivariate MR analyses to determine the independent causal impacts of seven metabolites in disease progress. Accordingly, the MR investigation revealed potential causal links between blood metabolites and PSC. It was found that genetically elevated levels of 1-stearoyl glycerophosphocholine (a subtype of LPC) promote PSC development, aligning with its role in promoting inflammation, which offers a new direction for early prevention and treatment of PSC. Moreover, two unknown metabolites have been confirmed by the MR Analysis to have a causal relationship with the PSC occurrence. Specifically, X-12405 was found to be causally associated with a higher risk of PSC, while X-11847 played a protective role in the development of PSC. Briefly, metabolic pathway analysis pinpointed seven significant metabolic pathways. These findings provide novel directions for clinical screening, preventive strategies, and precision therapeutics.

By developing a deeper understanding of the complex mechanisms underlying the onset and progression of cholestasis and leveraging powerful analytical methods, such as MR analysis, the opportunity arises to enable earlier detection and treatment of the disease for future generations. Meanwhile, further investigations are required to elucidate the specific mechanisms and signaling pathways that link mannose to the PBC onset. The identification of these mechanisms and pathways is essential for the development of targeted therapies and interventions aimed at preventing or delaying the onset of PBC and mitigating its associated morbidity and mortality. As such, continued research in this area is of paramount importance for advancing our understanding of cholestasis and improving patient outcomes.

## Data availability statement

Publicly available datasets were analyzed in this study. This data can be found here: the summary statistics for metabolites as exposure data were derived from one of the most comprehensive metabolomic genome-wide association studies (mGWAS) published by Shin et al. (10).

## Author contributions

ZxW: Writing – review & editing, Supervision, Project administration, Funding acquisition, Formal analysis, Conceptualization. YL: Writing – original draft, Visualization, Software, Methodology, Investigation. QX: Writing – original draft, Visualization, Validation, Resources, Investigation, Formal analysis. XM: Writing – original draft, Validation, Resources, Investigation, Data curation, Conceptualization. JL: Writing – original draft, Visualization, Validation, Software, Methodology, Formal analysis. ZjW: Writing – original draft, Methodology, Investigation, Formal analysis, Data curation.

## Footnotes

^1^ https://www.mrbase.org/

^2^ http://www.phenoscanner.medschl.cam.ac.uk/

^3^ https://www.metaboanalyst.ca/
