# Supplementary material for: Causality of metabolites and metabolic pathways on cholestatic liver diseases: a Mendelian randomization study
Source: Front Med (Lausanne). 2024 Jul 2;11:1395526. doi: 10.3389/fmed.2024.1395526 (PMC11250271; doi:10.3389/fmed.2024.1395526)
Supplement: Supplementary file 1 [file Data_Sheet_1.docx]

**Supplementary Material**

**Figure S1:** Scatterplot for the significant Mendelian randomization (MR) association (FDR < 0.05) between metabolites and 2 cholestatic liver diseases (PBC and PSC). SNP, single nucleotide polymorphism; PBC, primary biliary cholangitis; PSC, primary sclerosing cholangitis.

Within each panel, the black points represent the causal estimate of the association between a specific SNP and one of the cholestatic liver diseases (PBC and PSC). Each scatter point represents the MR estimation results of a SNP-disease pair. The lines in the plot indicate the overall estimates of the causal effect between the metabolite and the disease using different MR methods.

**Figure S2：** Forest plots for the Mendelian randomization (MR) leave-one-out analysis of the significant inverse variance weighted (IVW) estimates. single nucleotide polymorphism; PBC, primary biliary cholangitis; PSC, primary sclerosing cholangitis.

Within each panel, the black points represent the causal estimate of the association between a specific metabolite and 2 cholestatic liver diseases (PBC and PSC) after discarding each SNP in turn. Red points represent the pooled IVW estimates. Horizontal lines denote 95% confidence intervals.


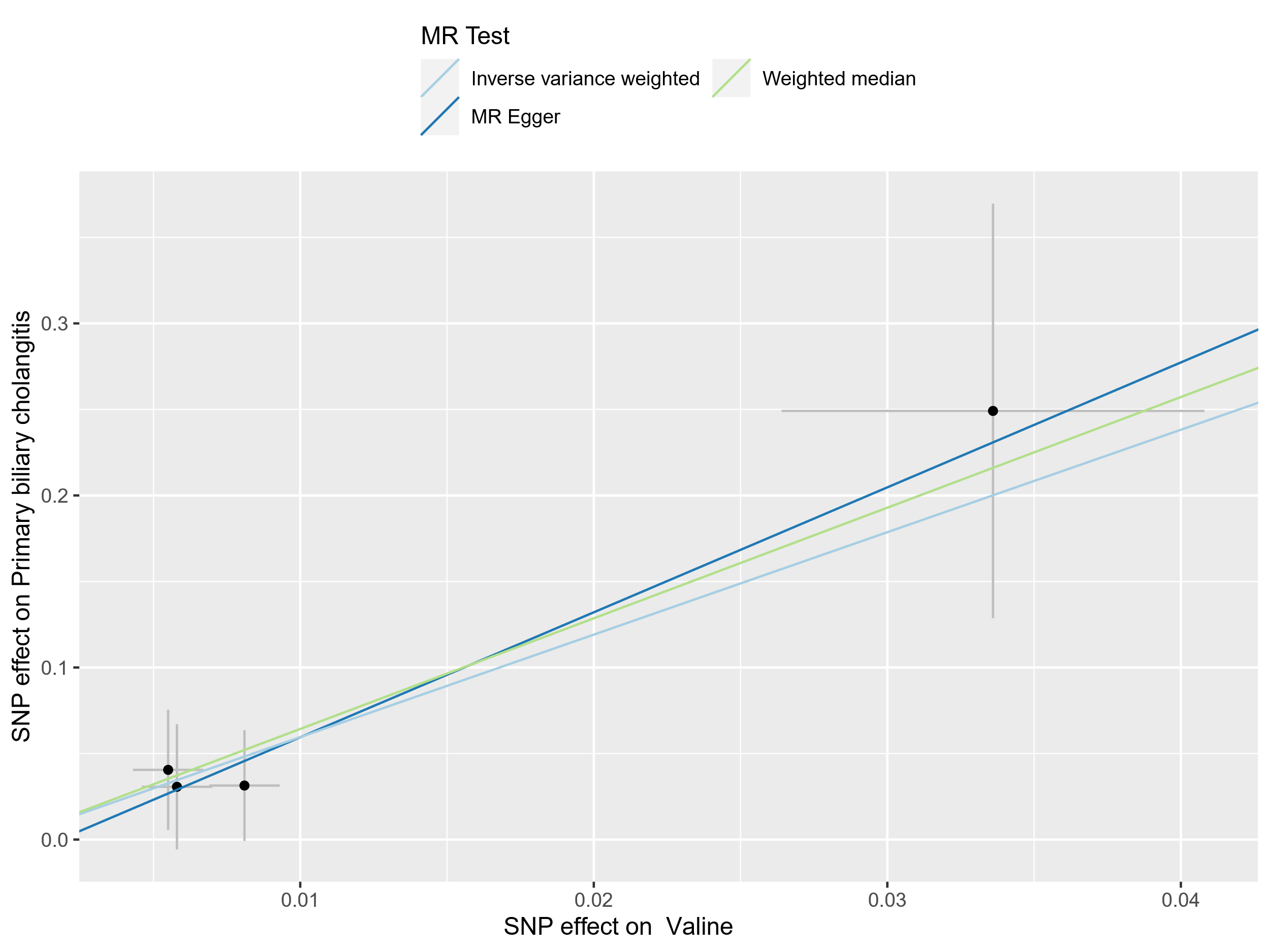

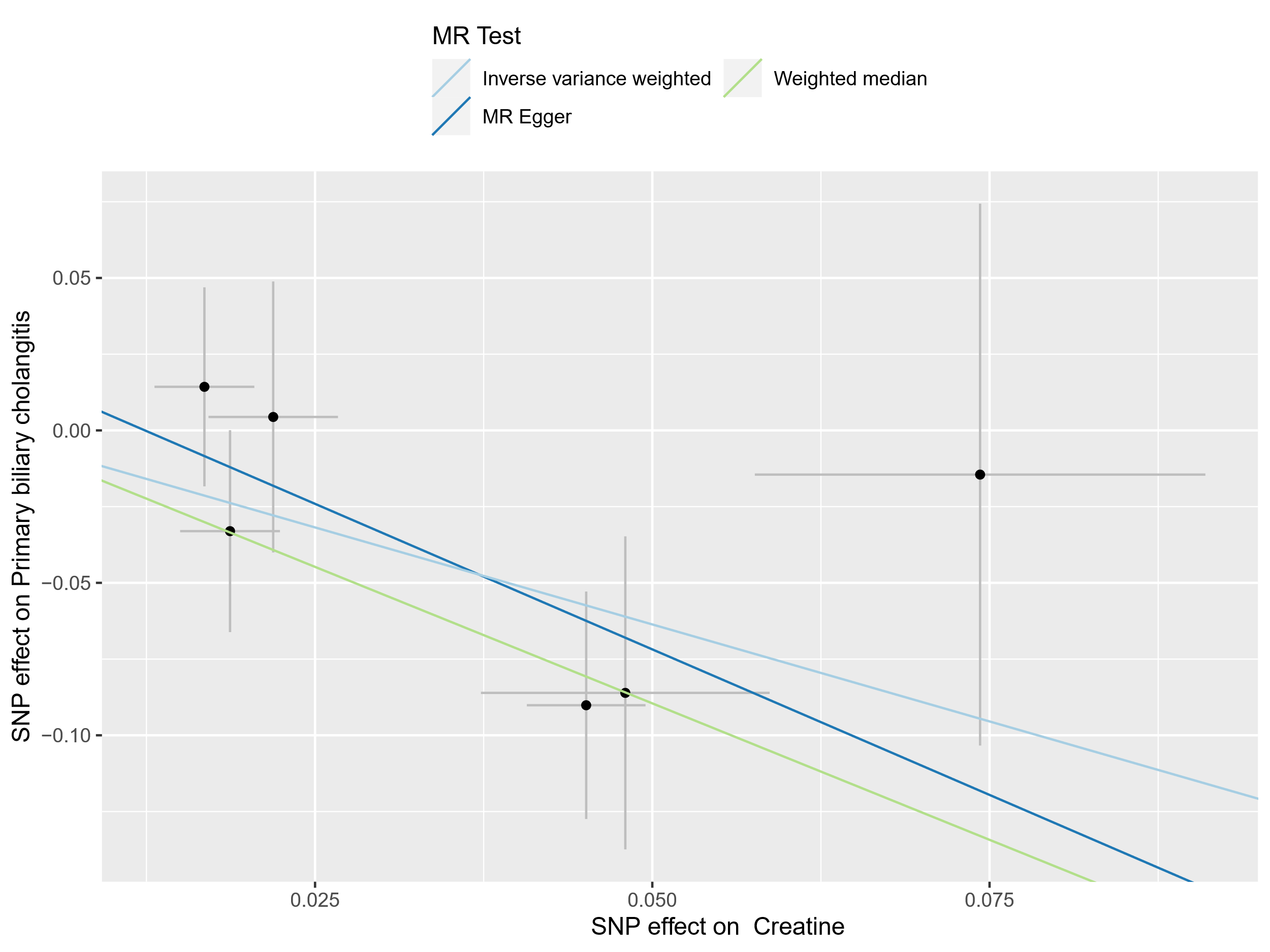

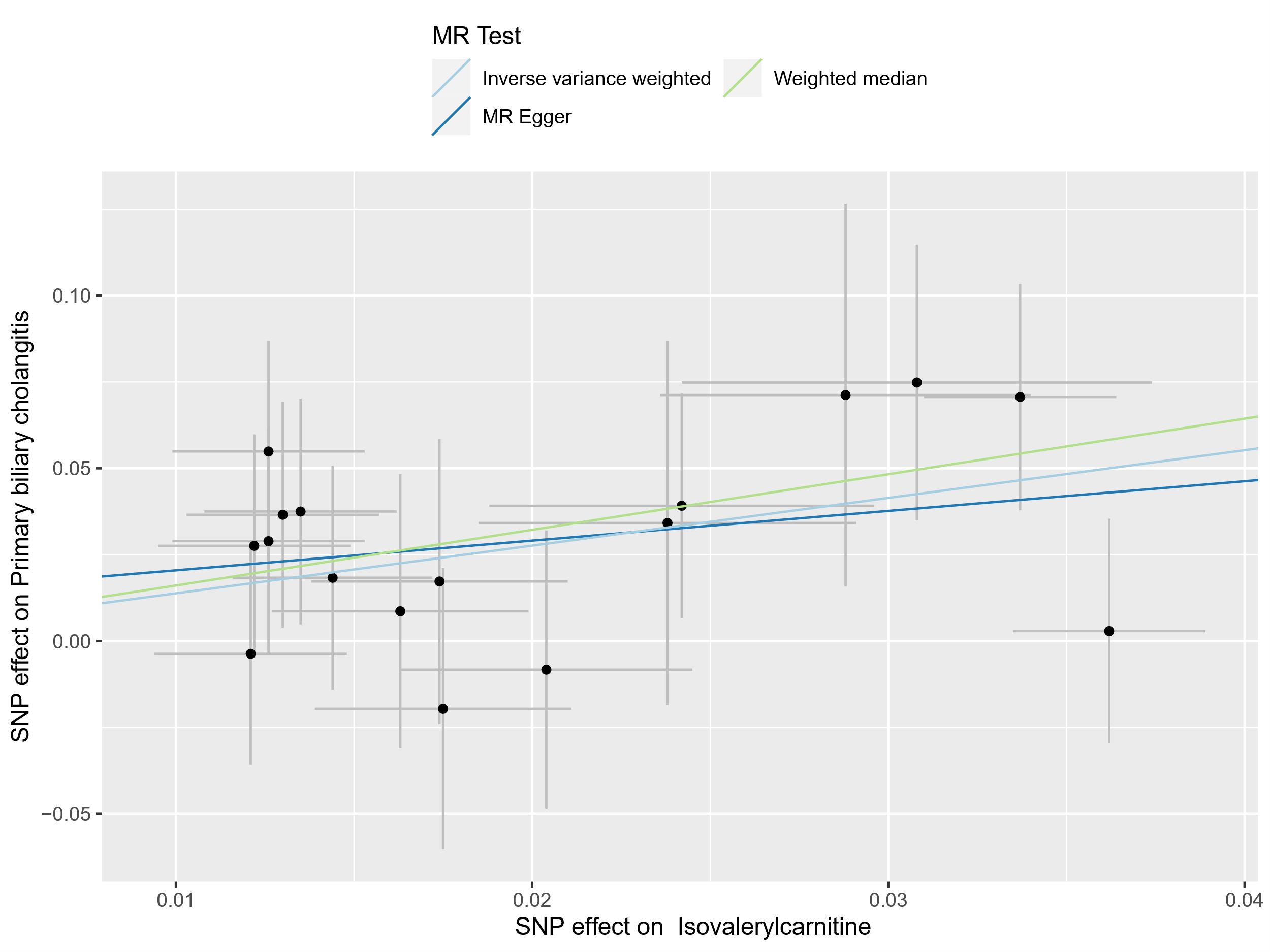

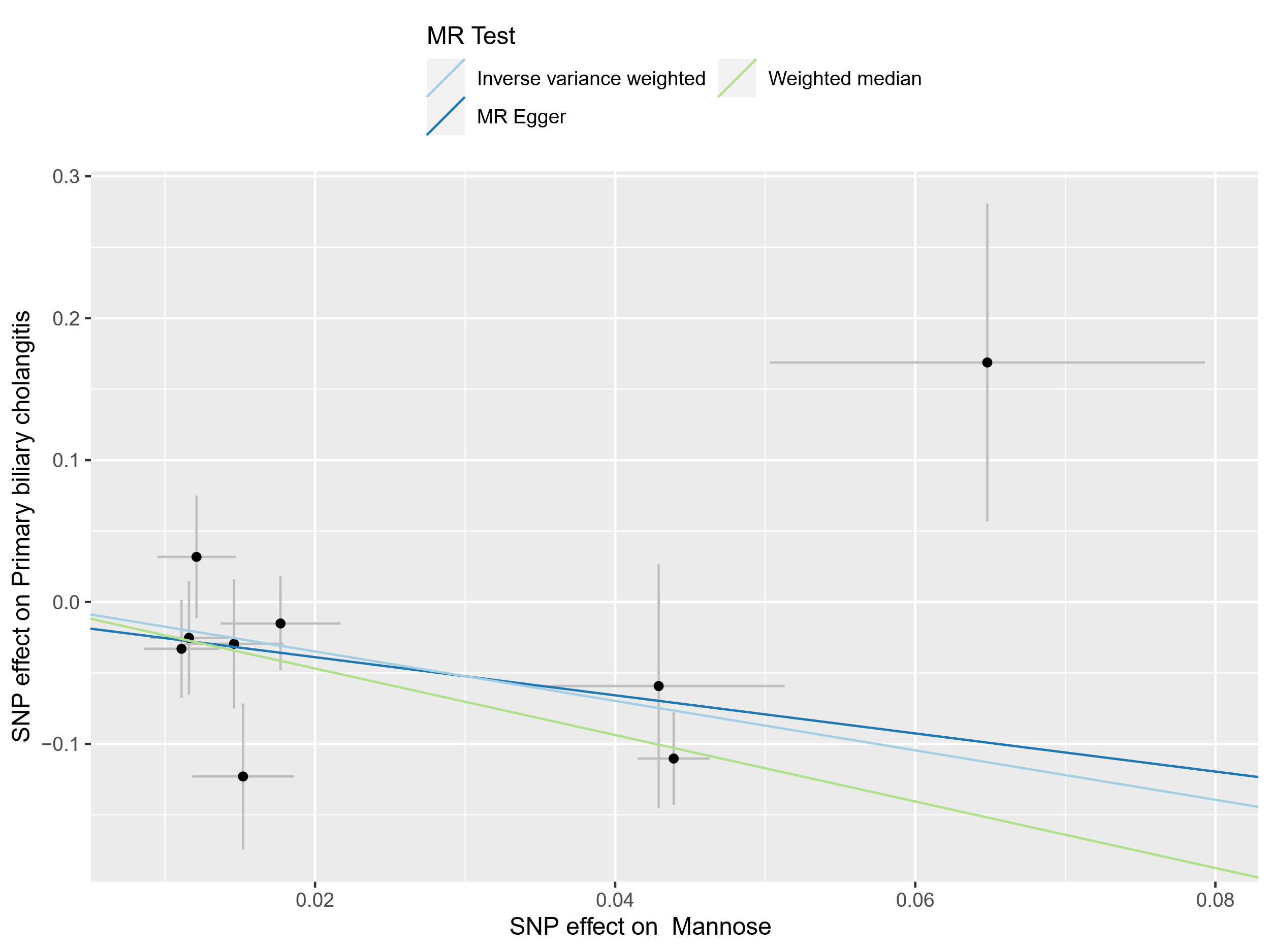

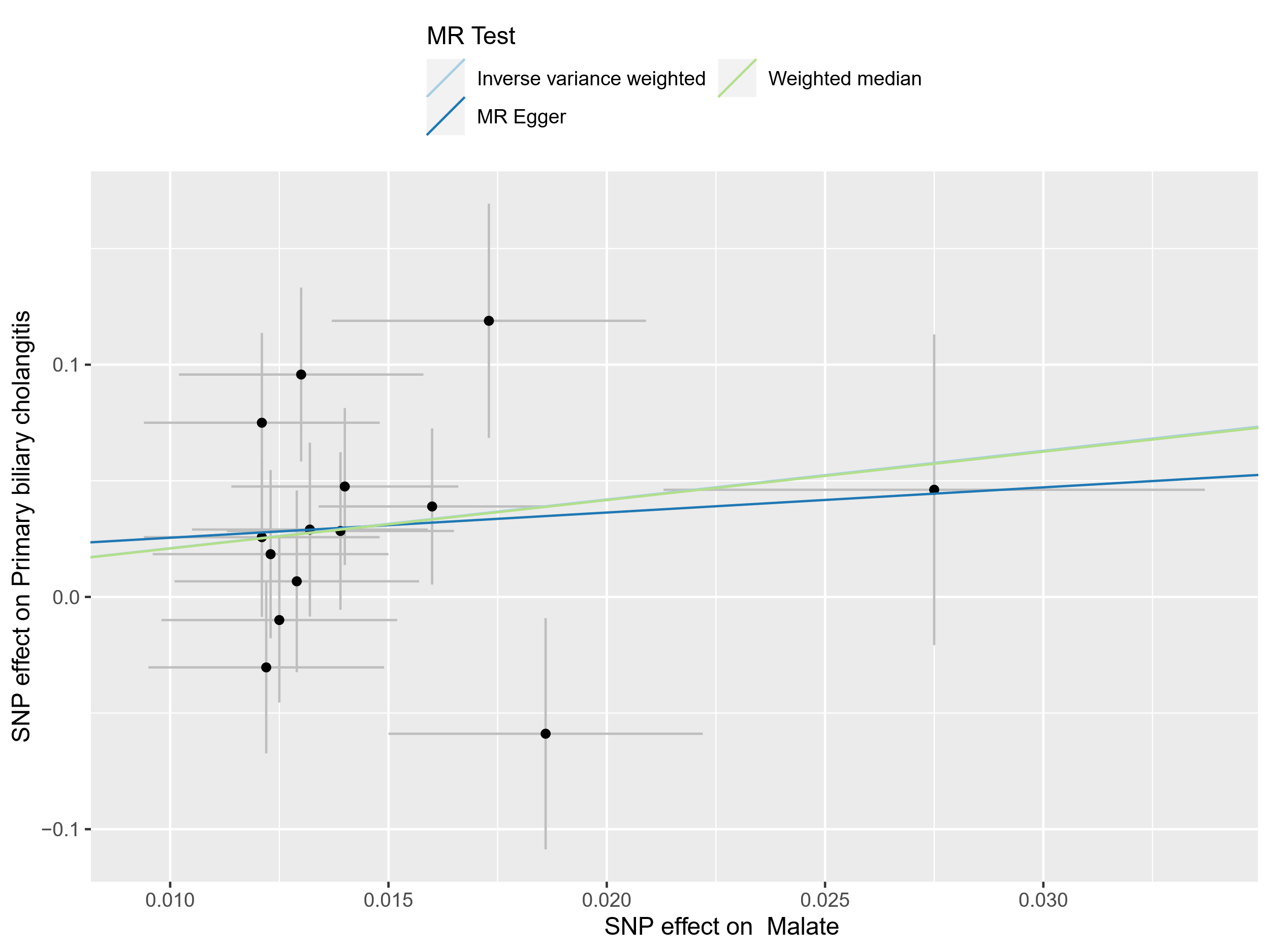

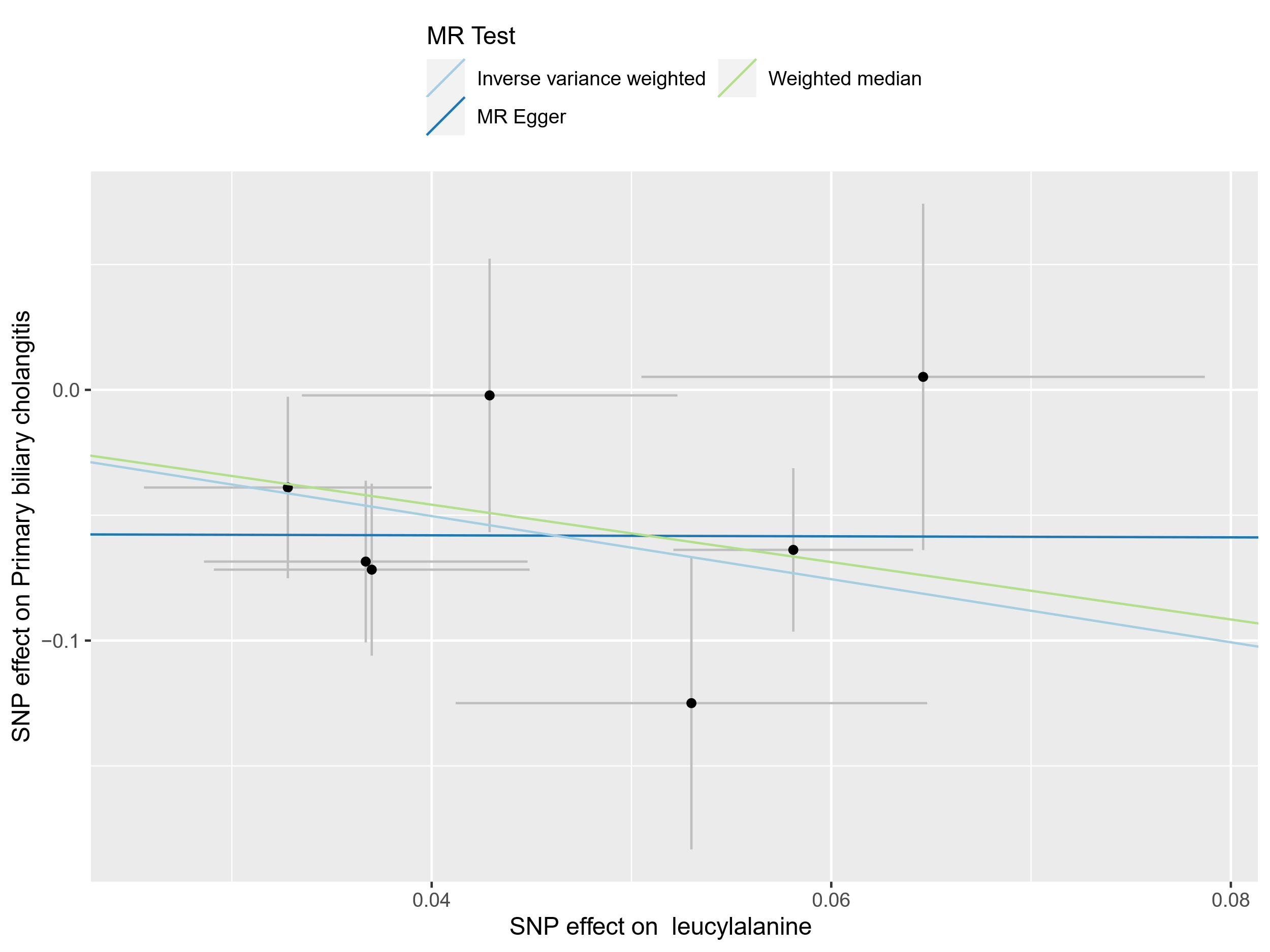

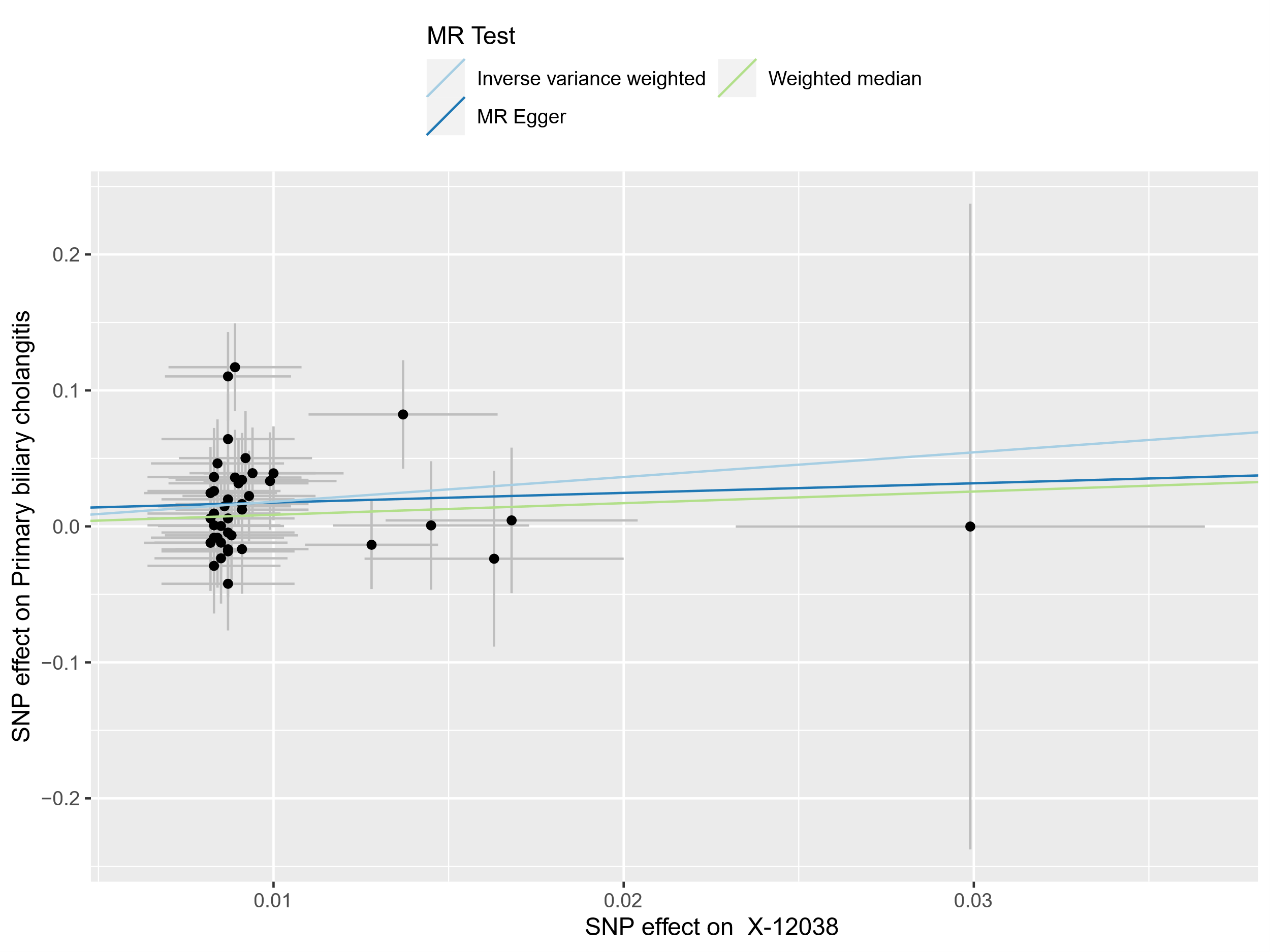


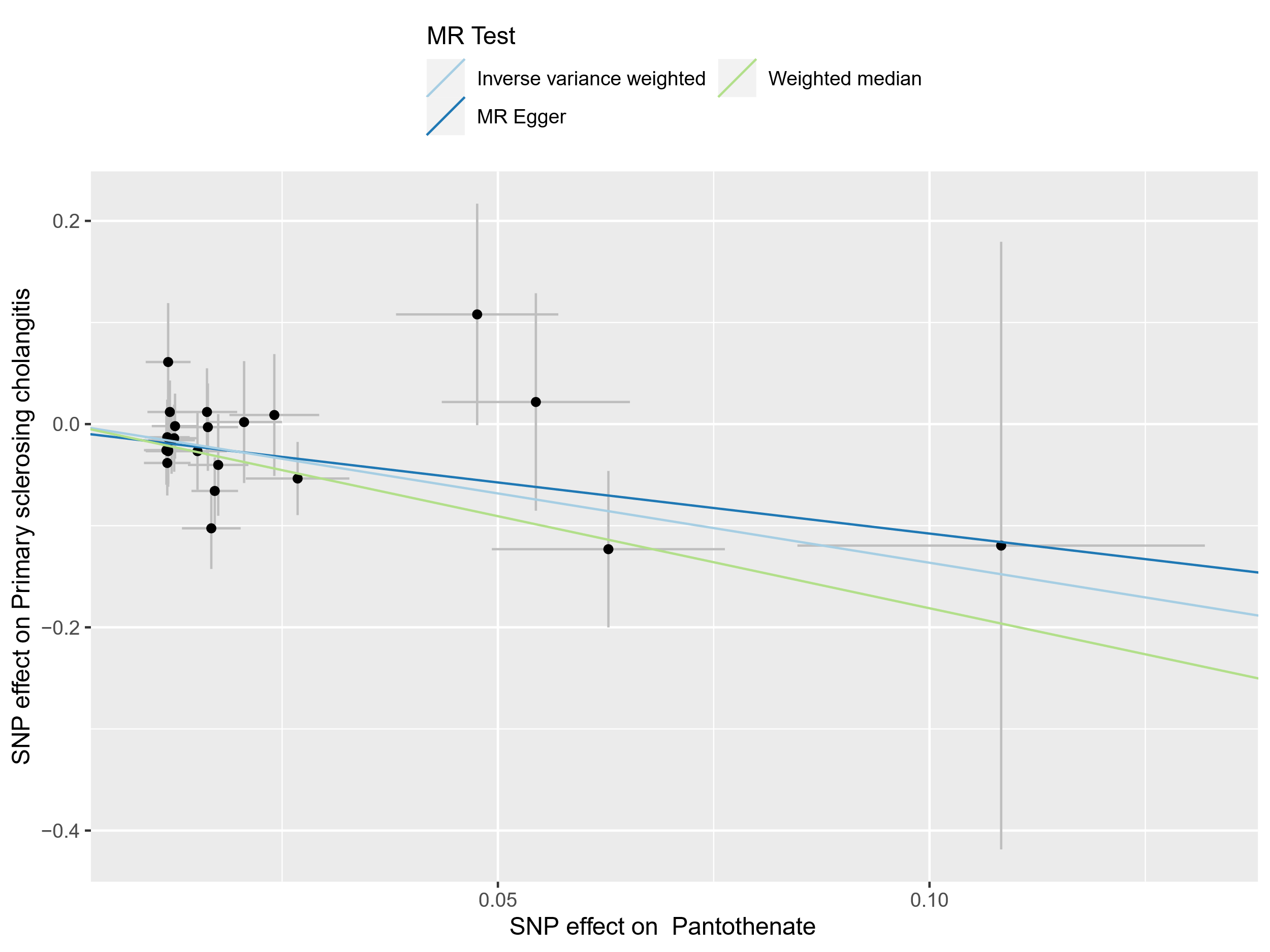

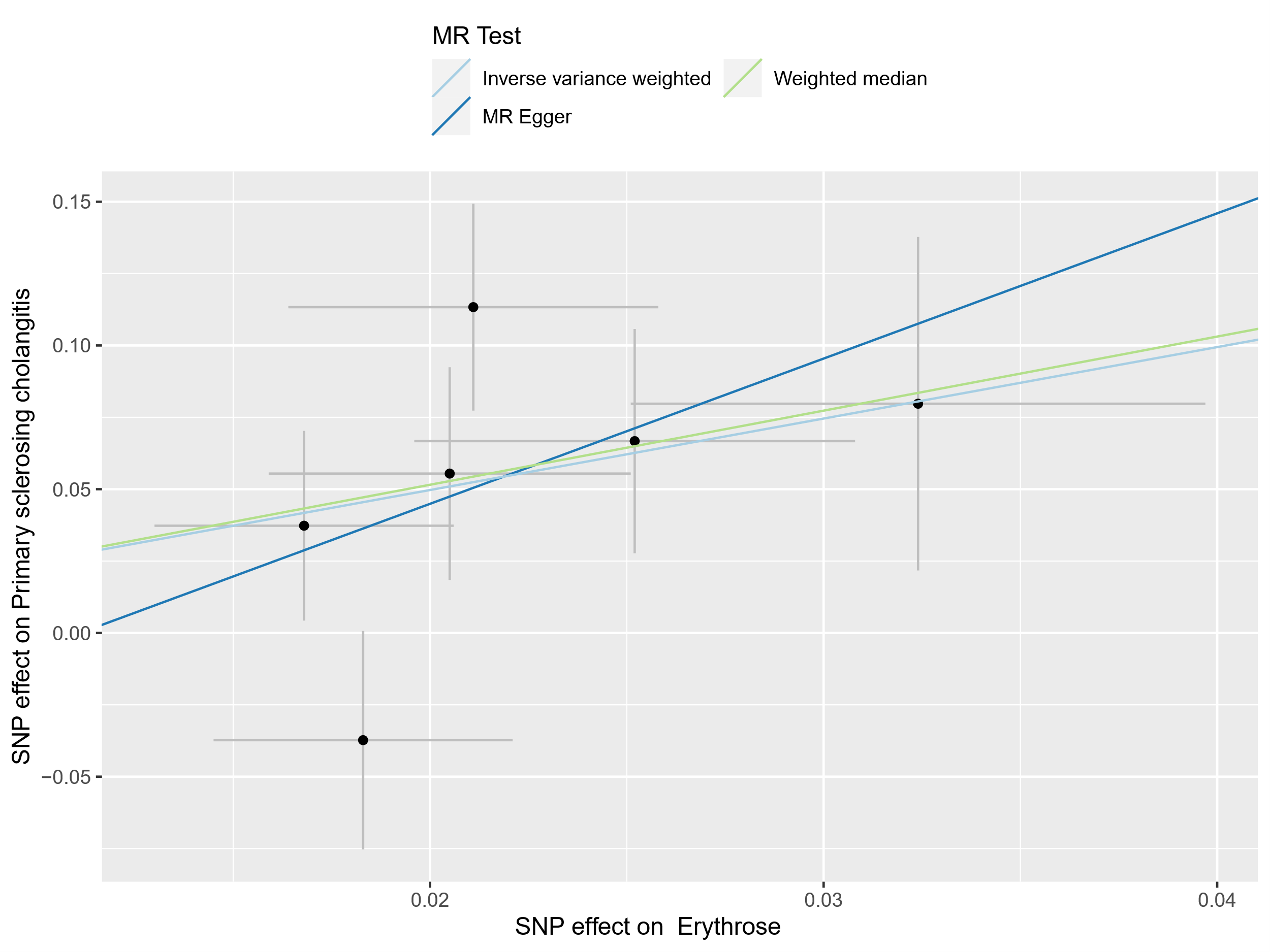

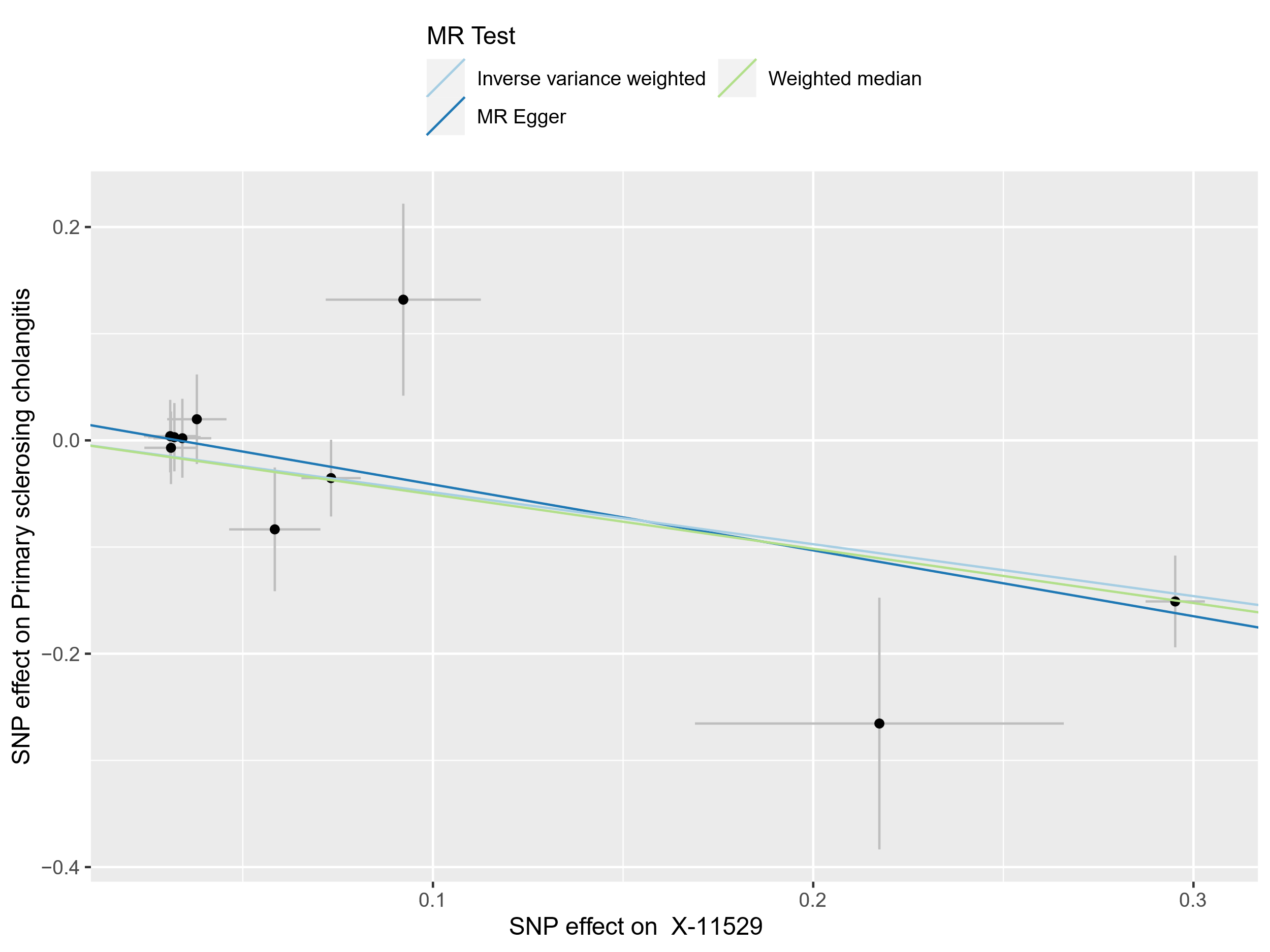

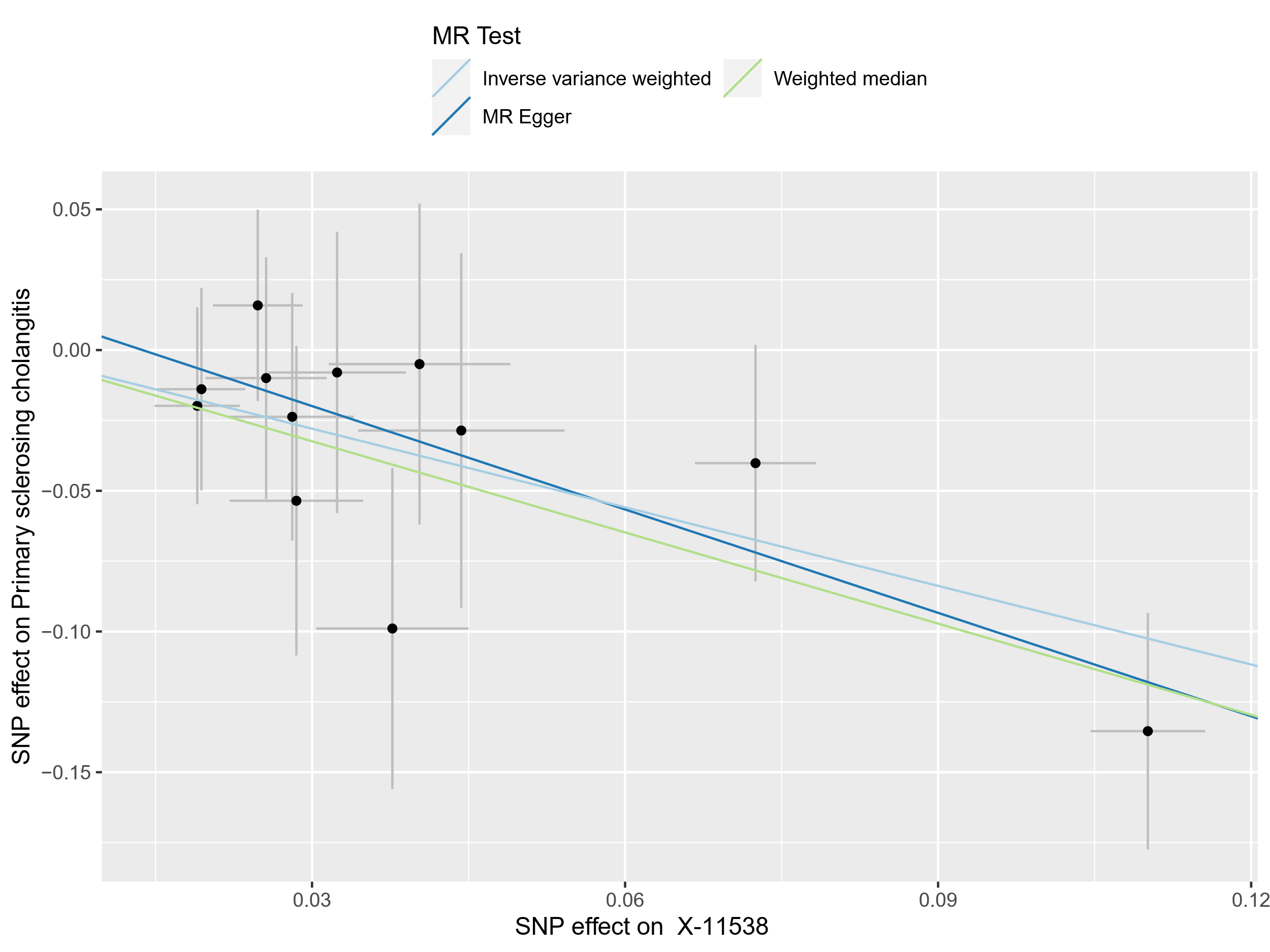

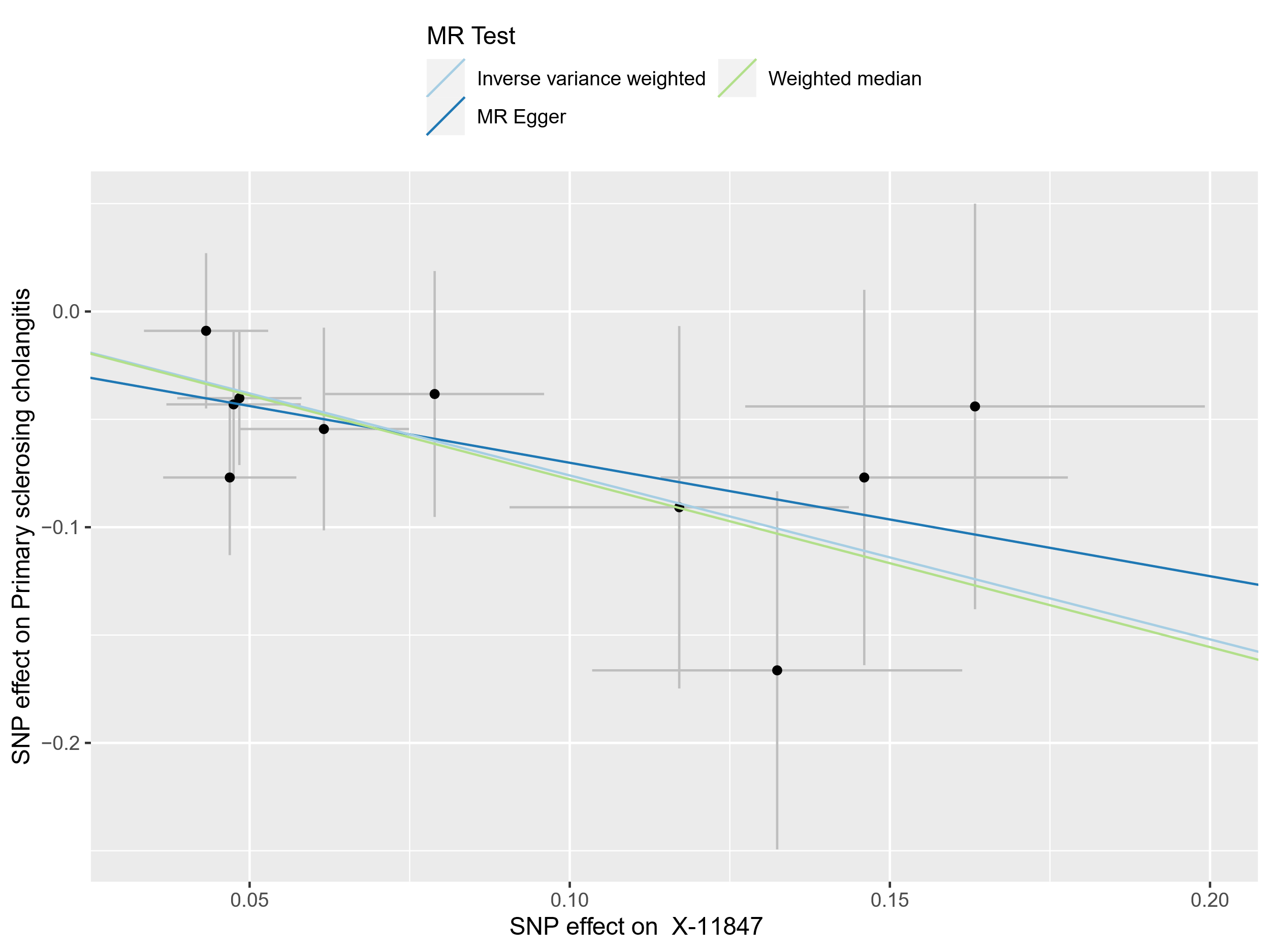

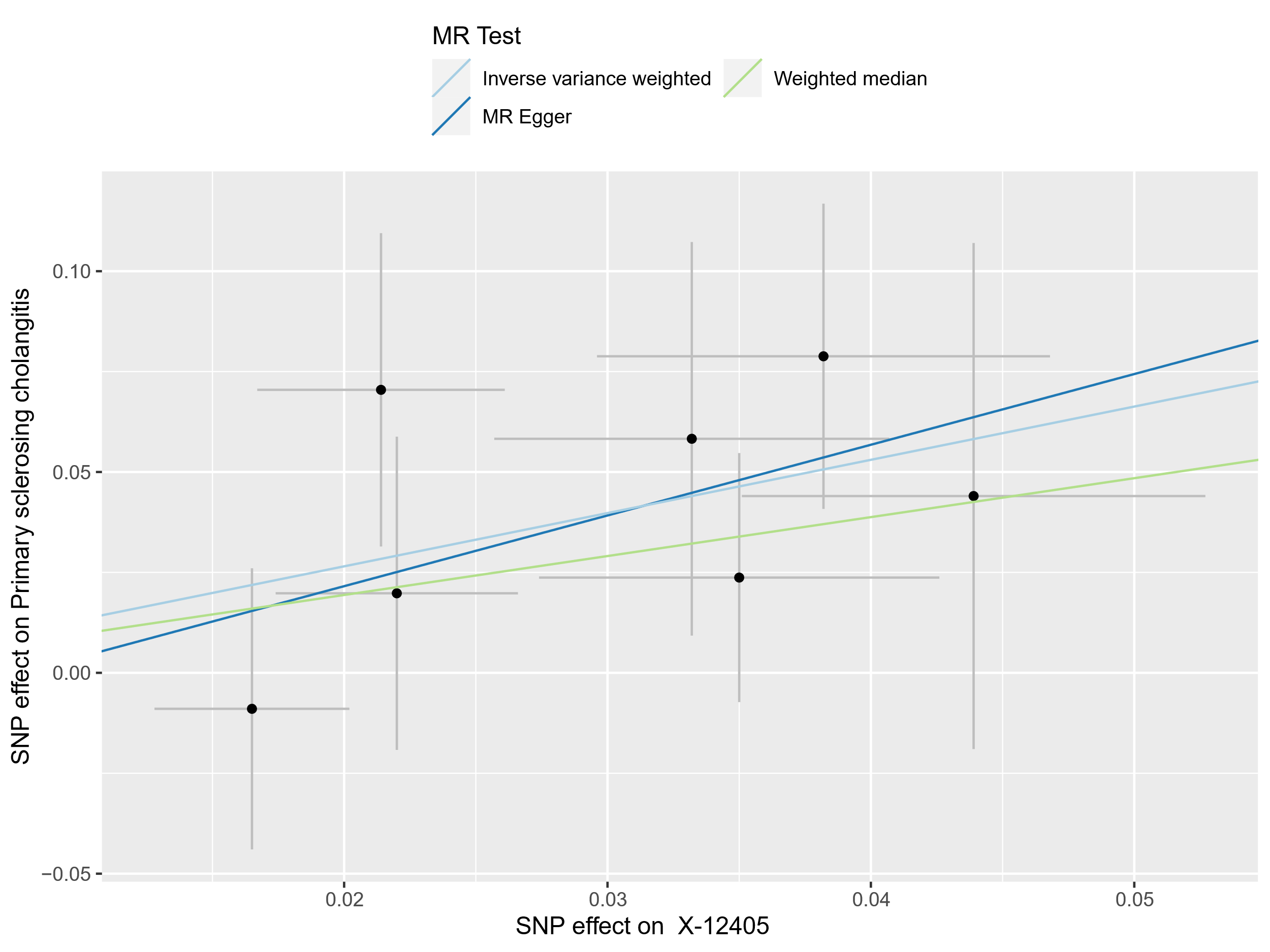

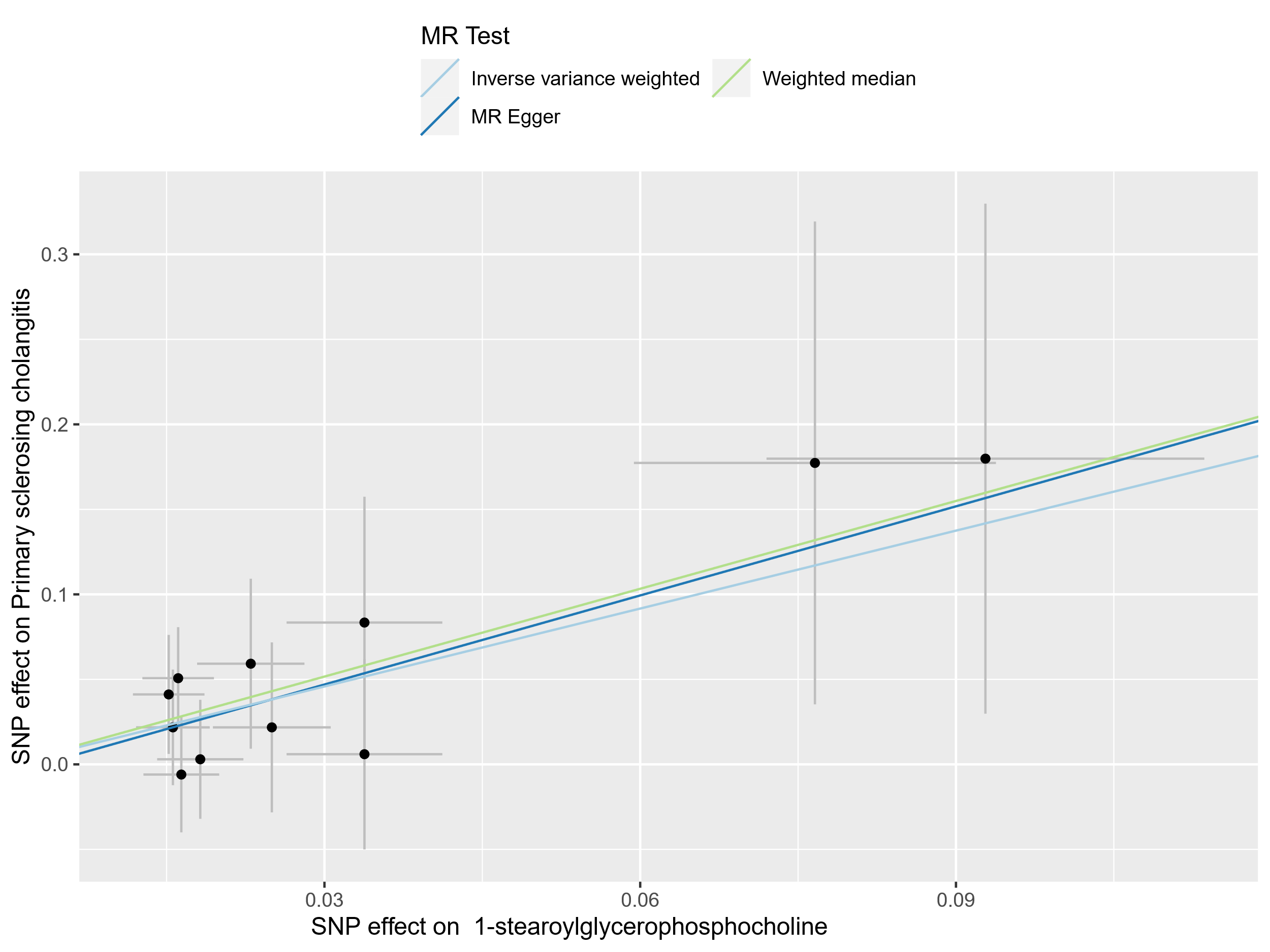

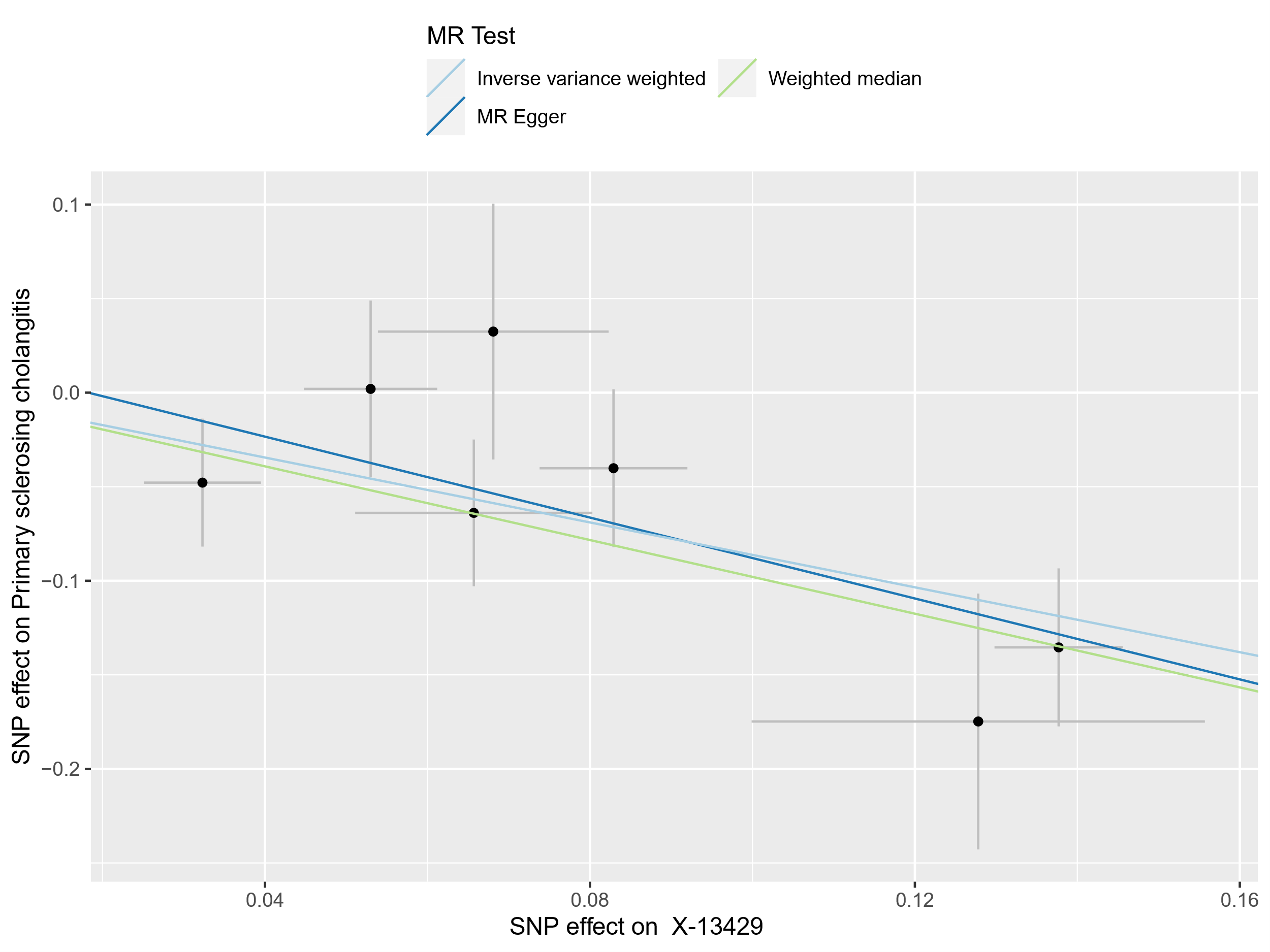


**Figure S1:** Scatterplot for the significant Mendelian randomization (MR) association (FDR < 0.05) between metabolites and 2 cholestatic liver diseases (PBC and PSC). SNP, single nucleotide polymorphism; PBC, primary biliary cholangitis; PSC, primary sclerosing cholangitis.


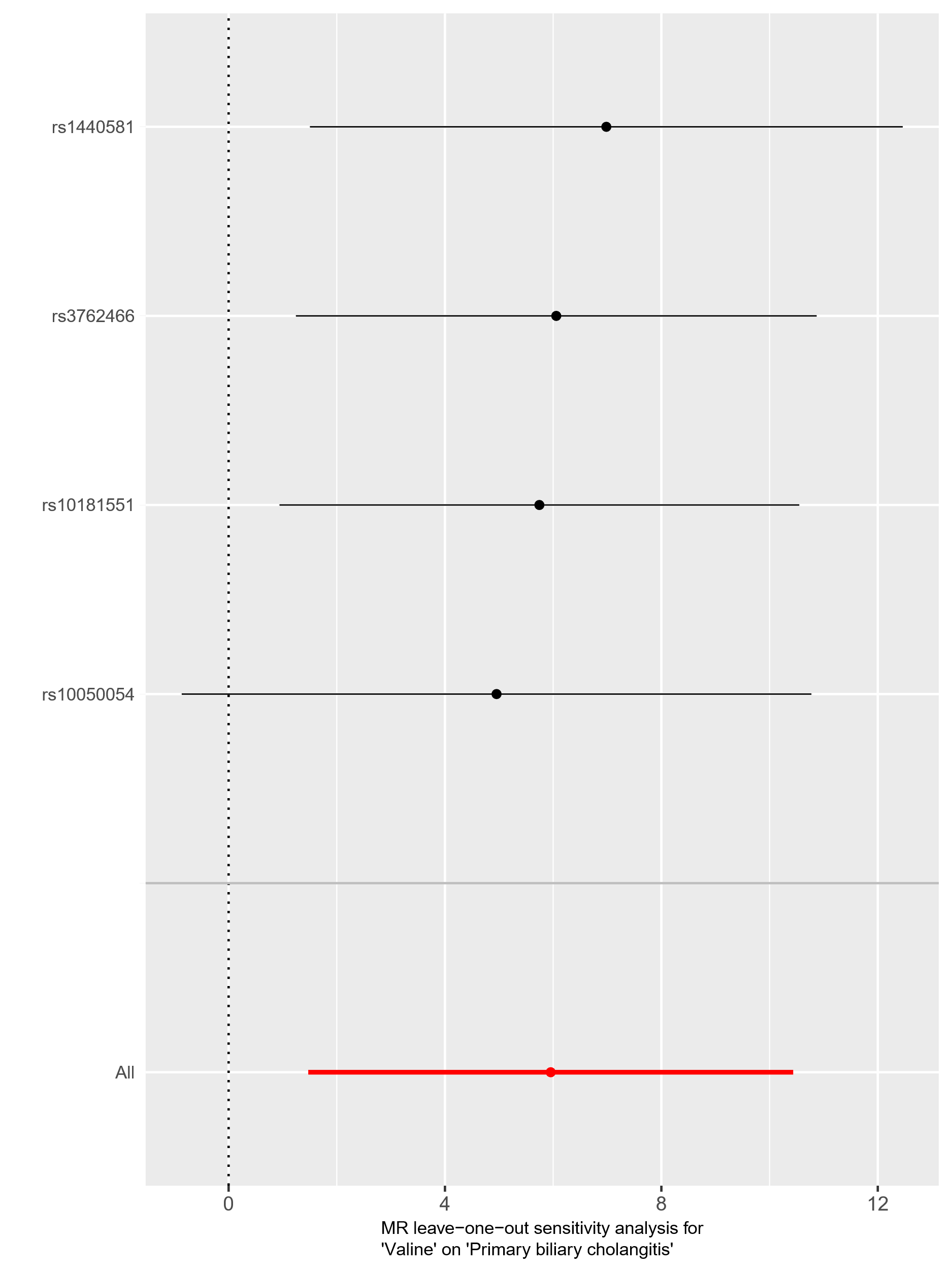

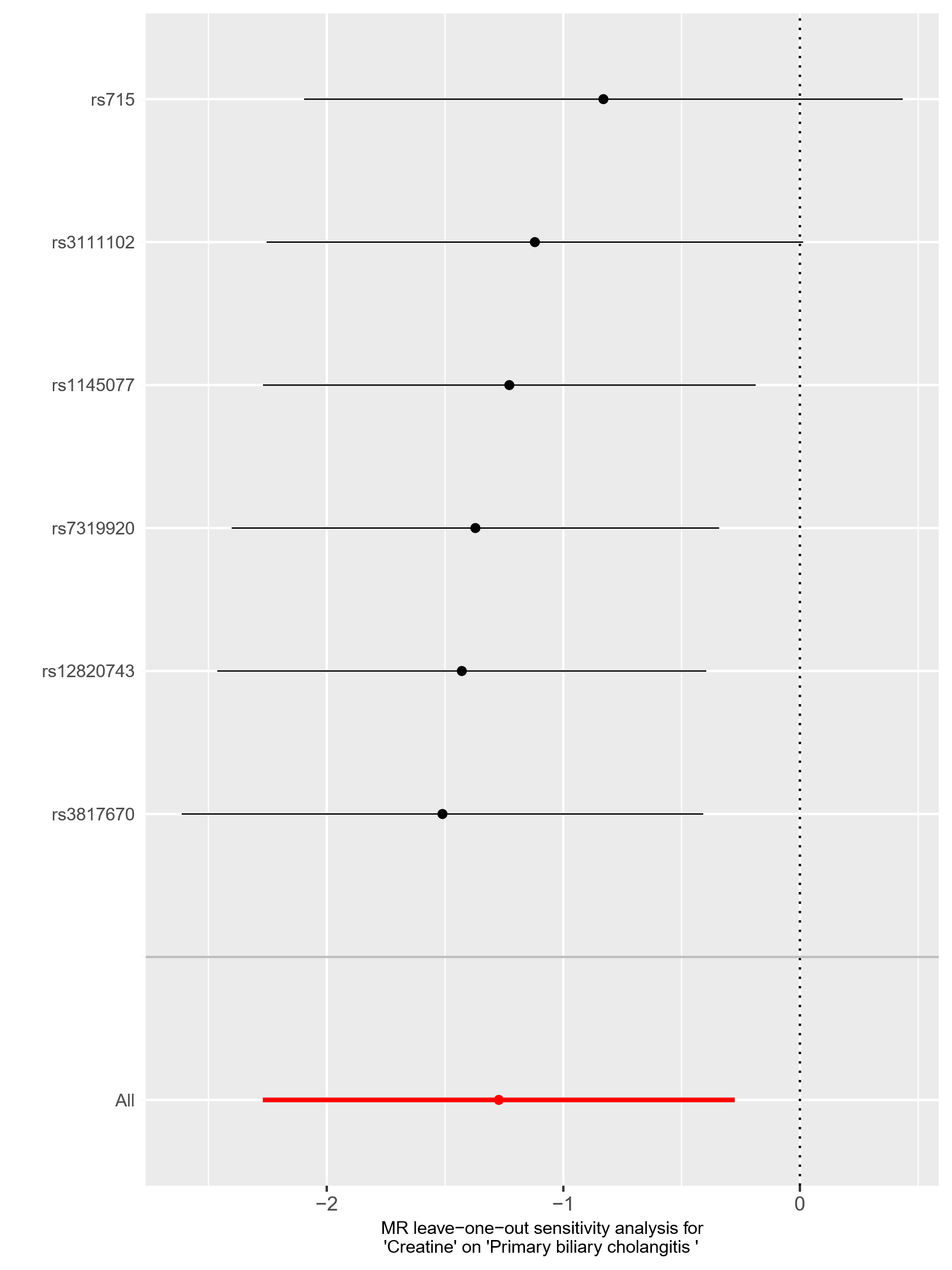

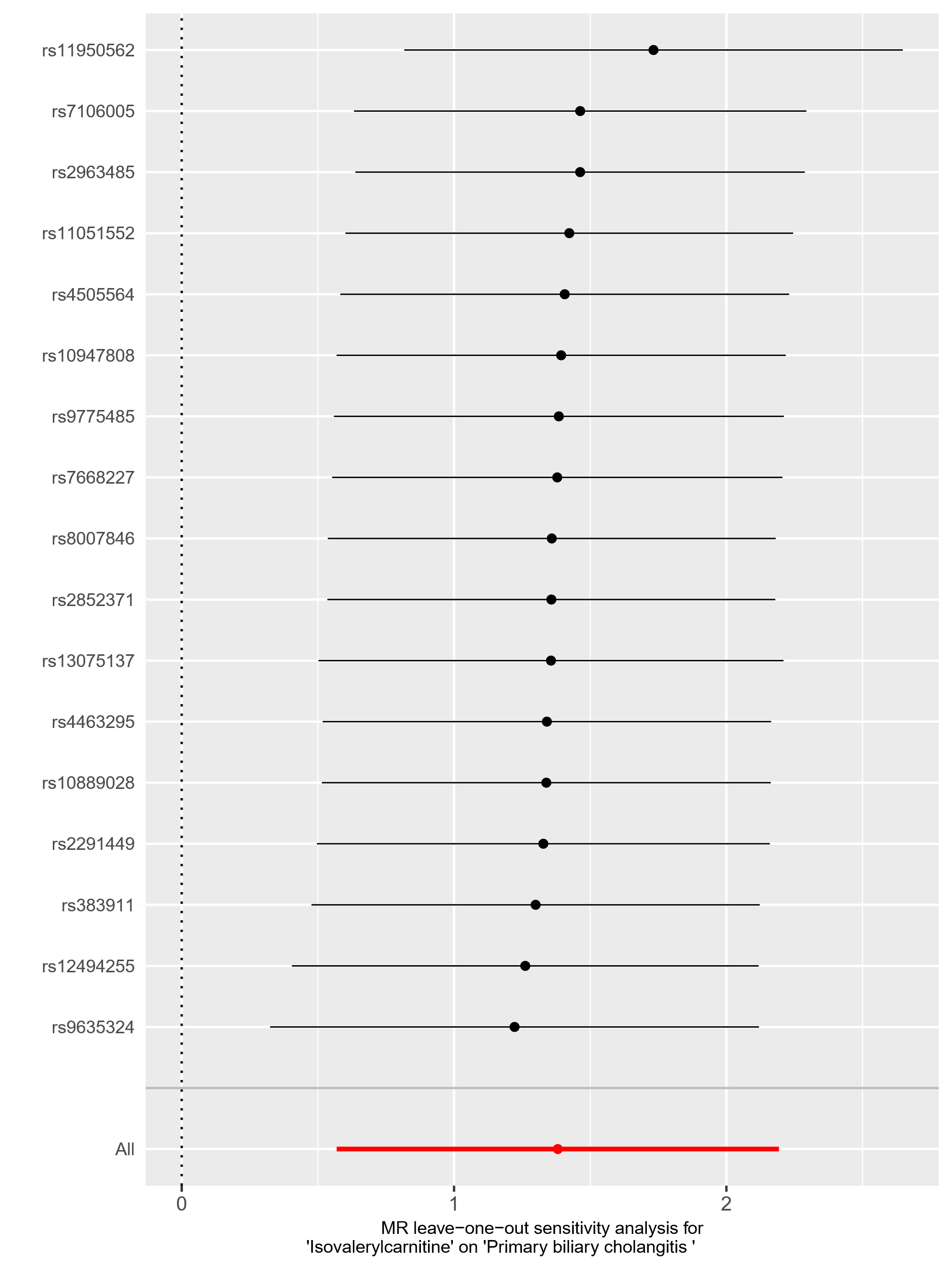

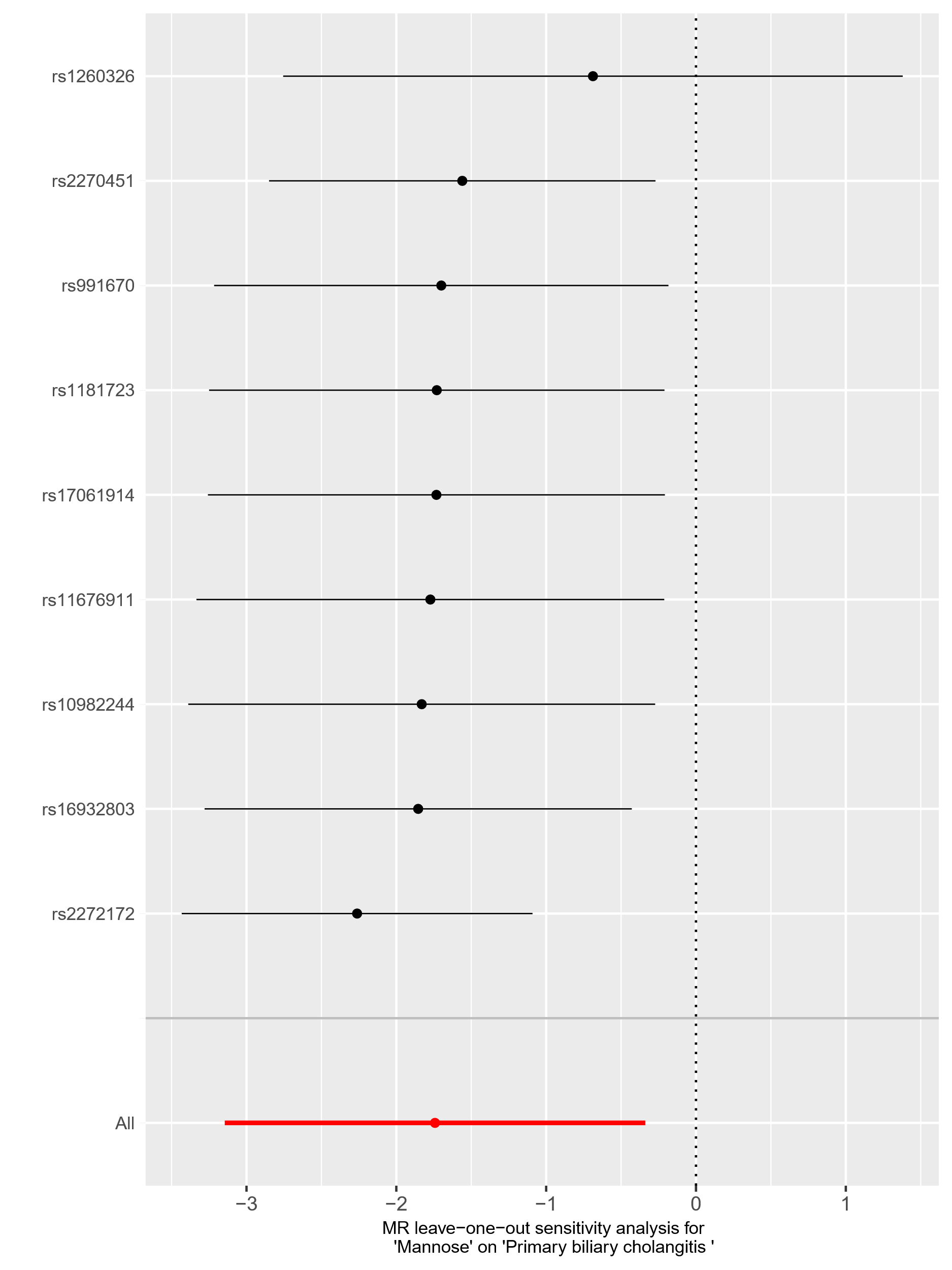

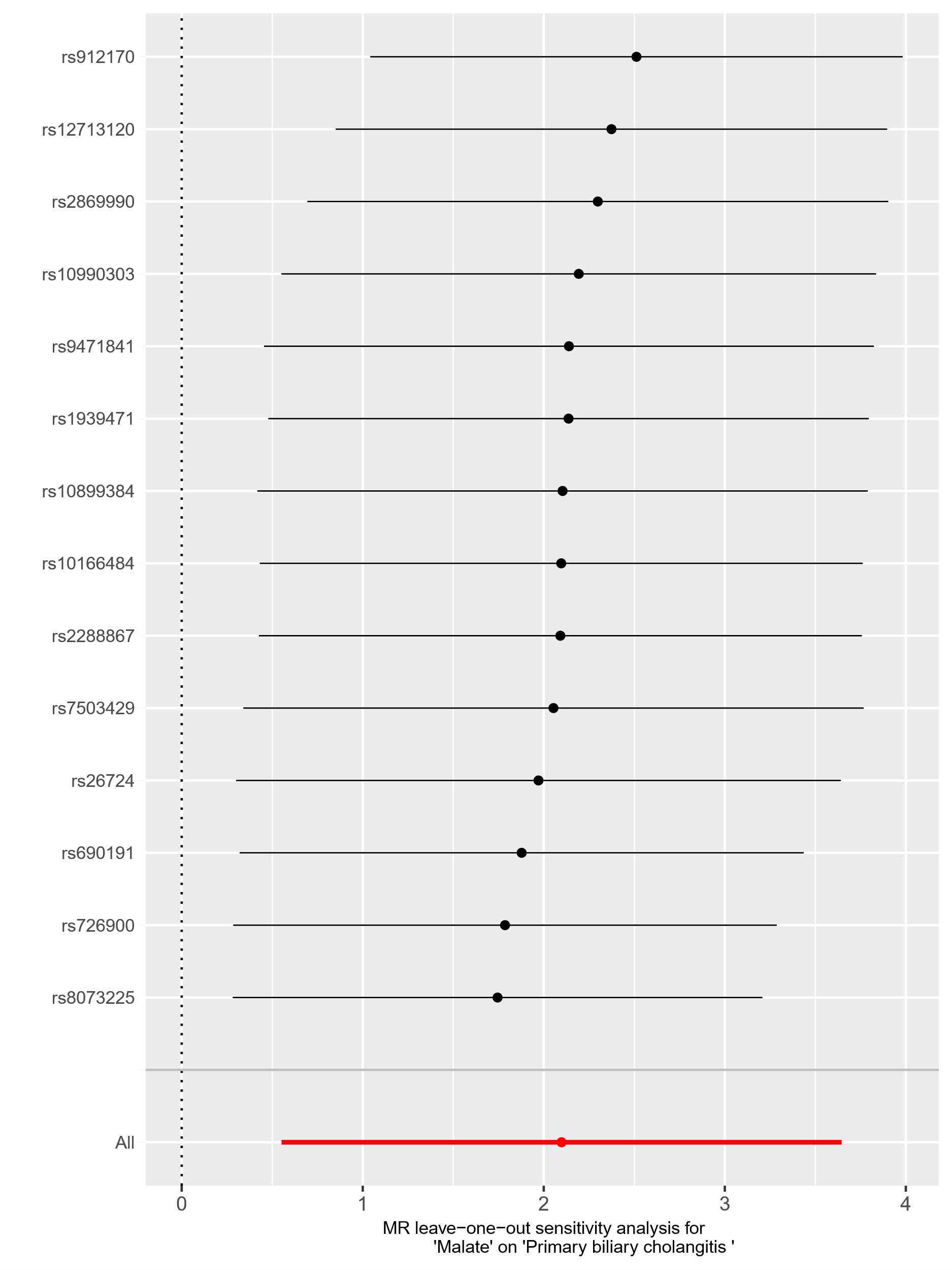

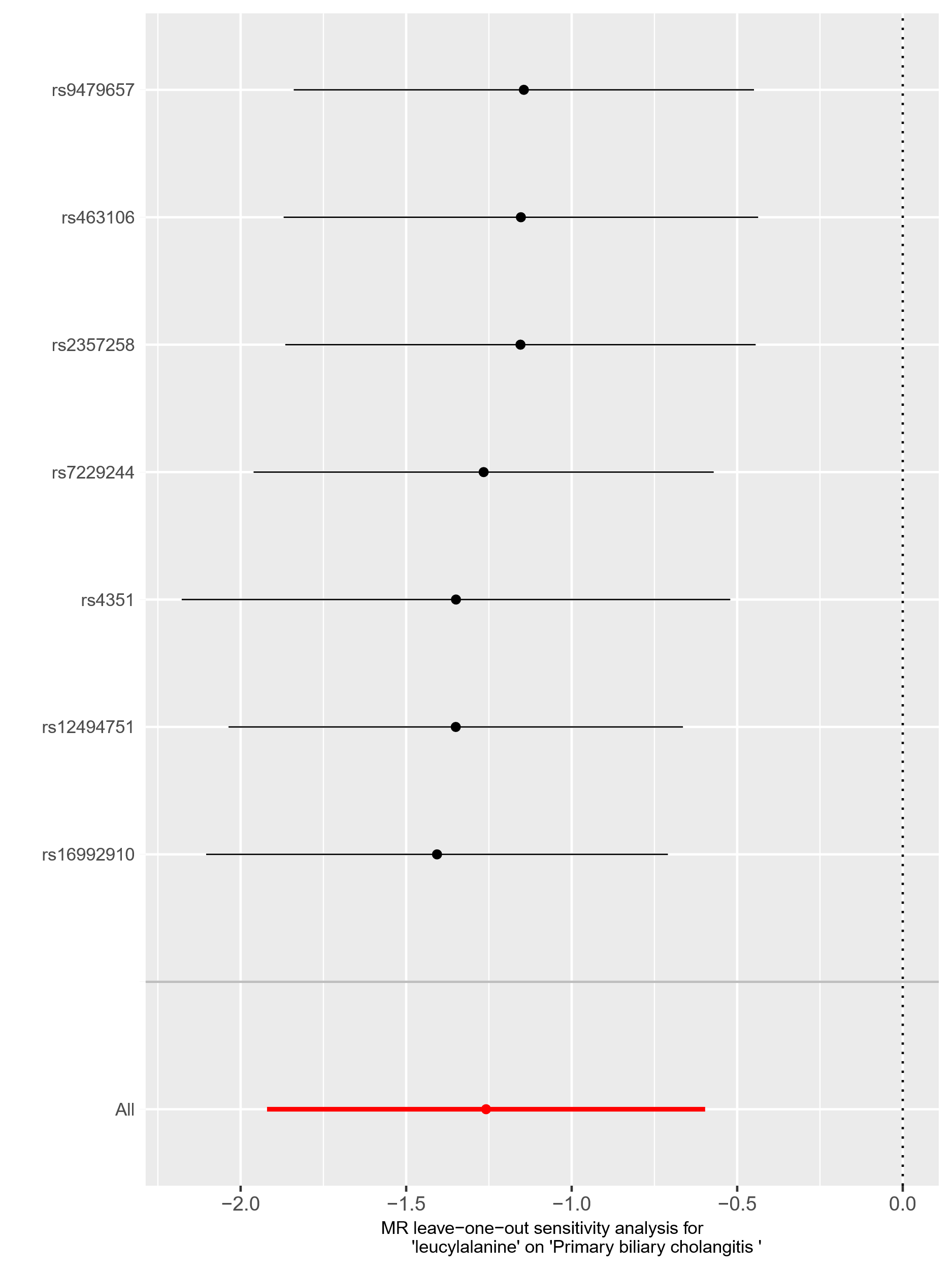

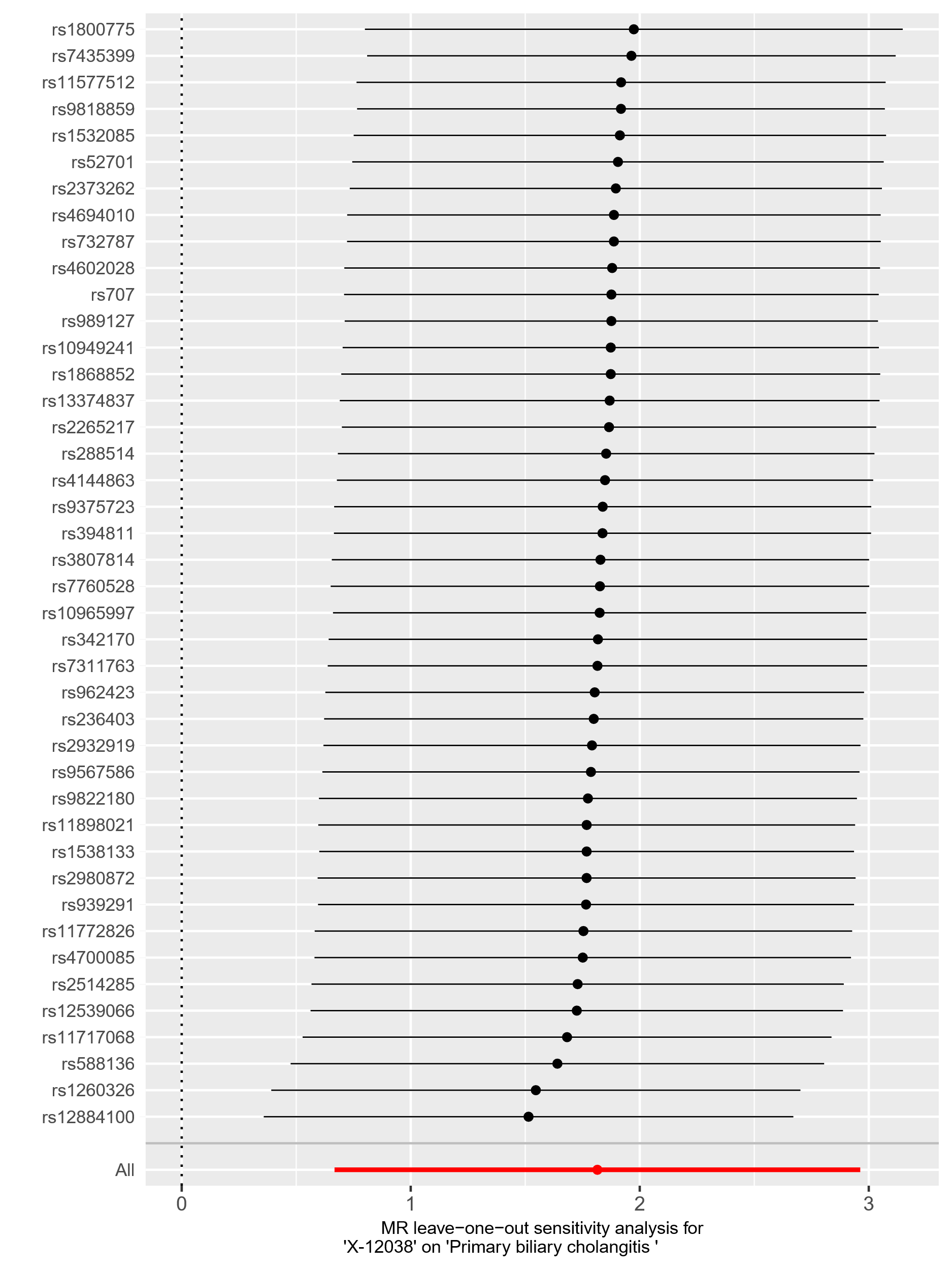


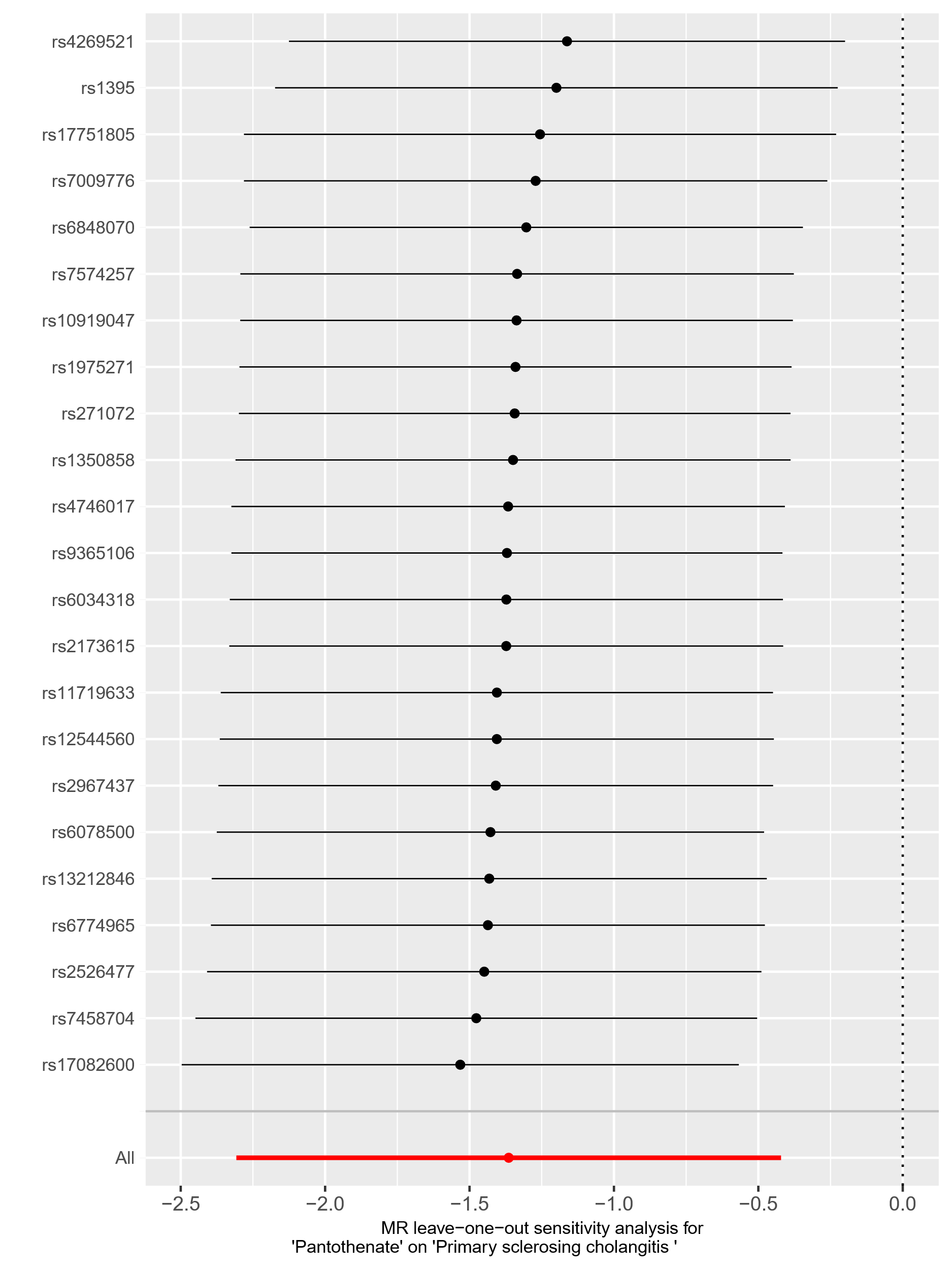

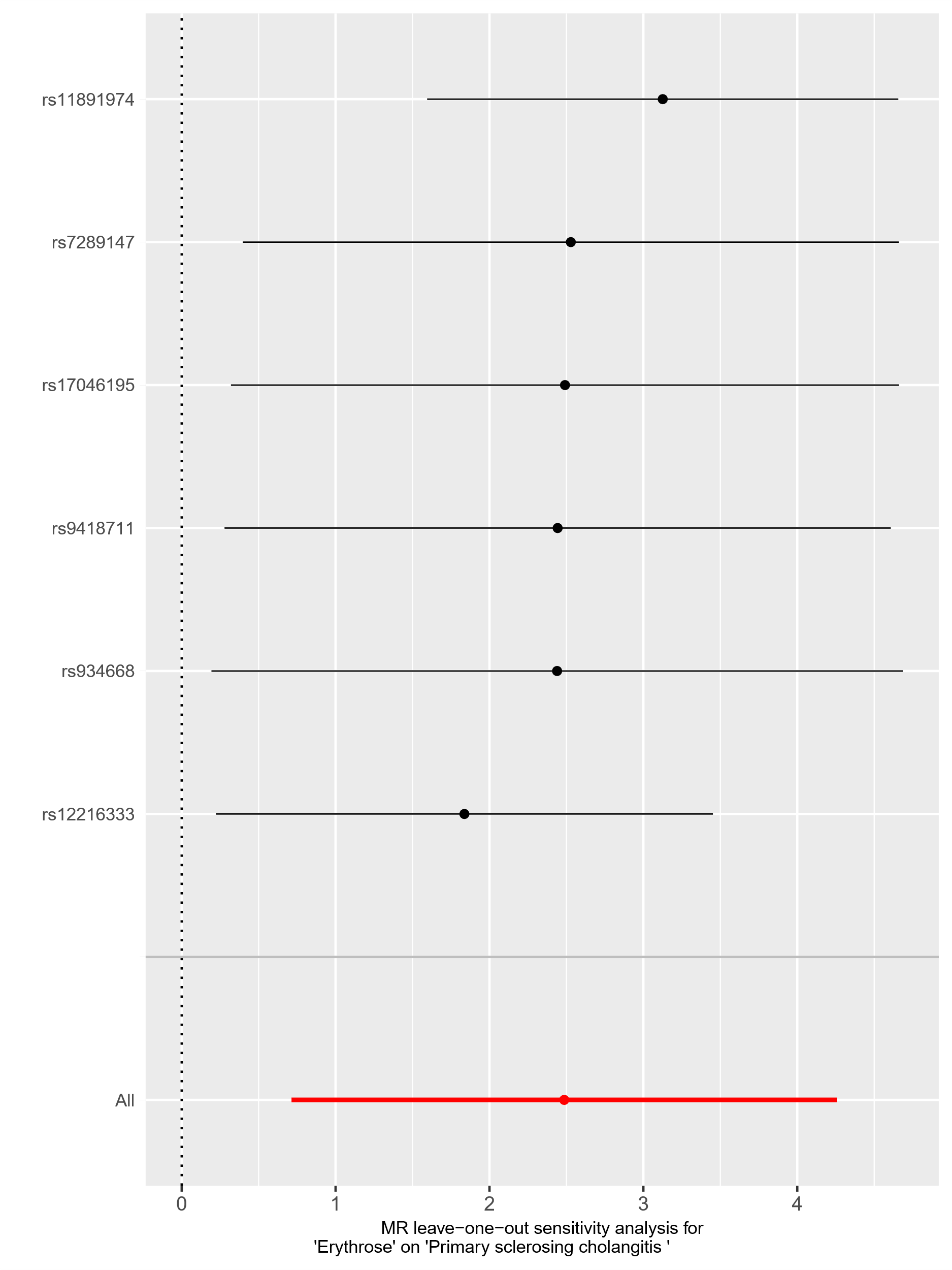

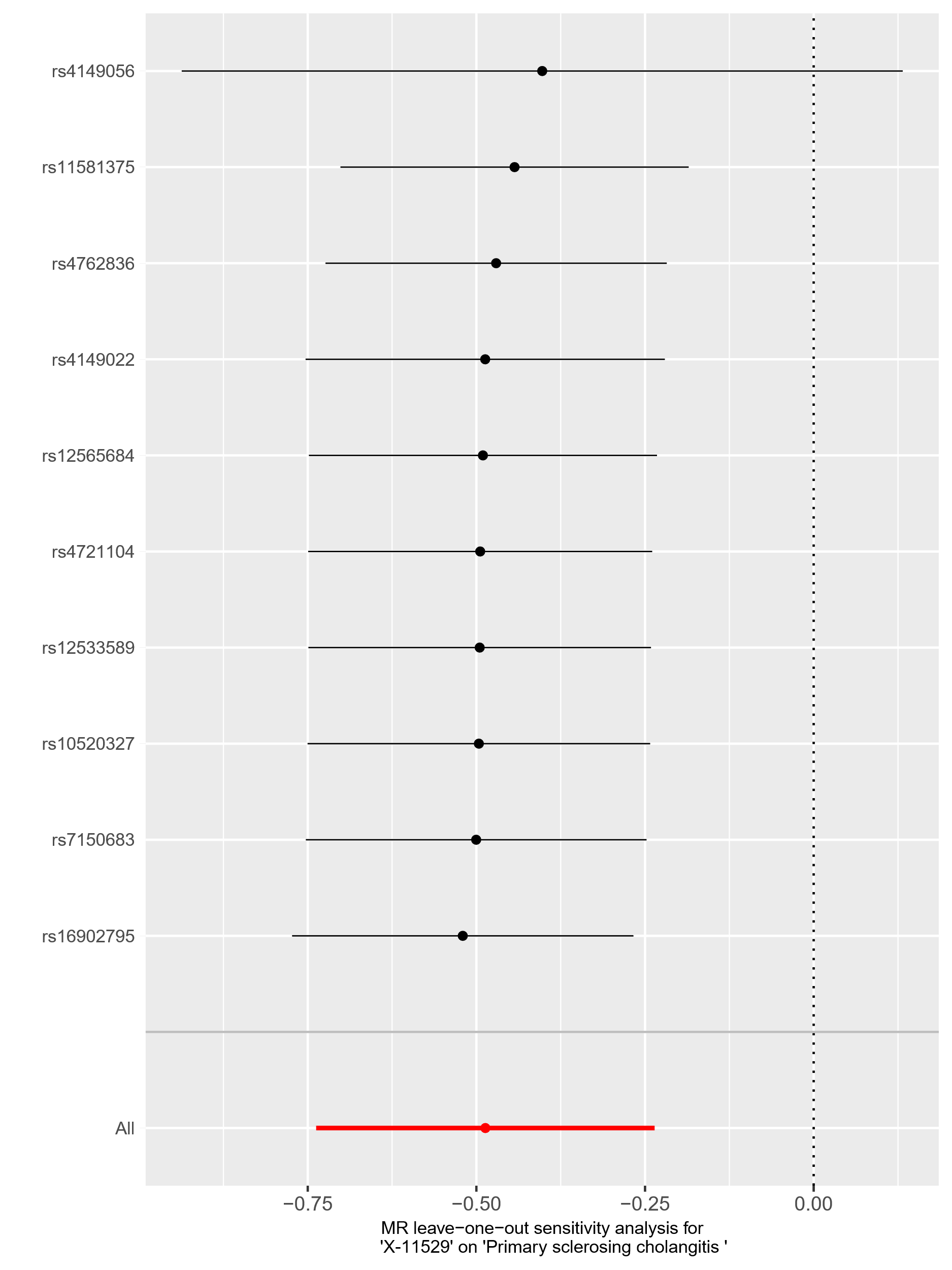

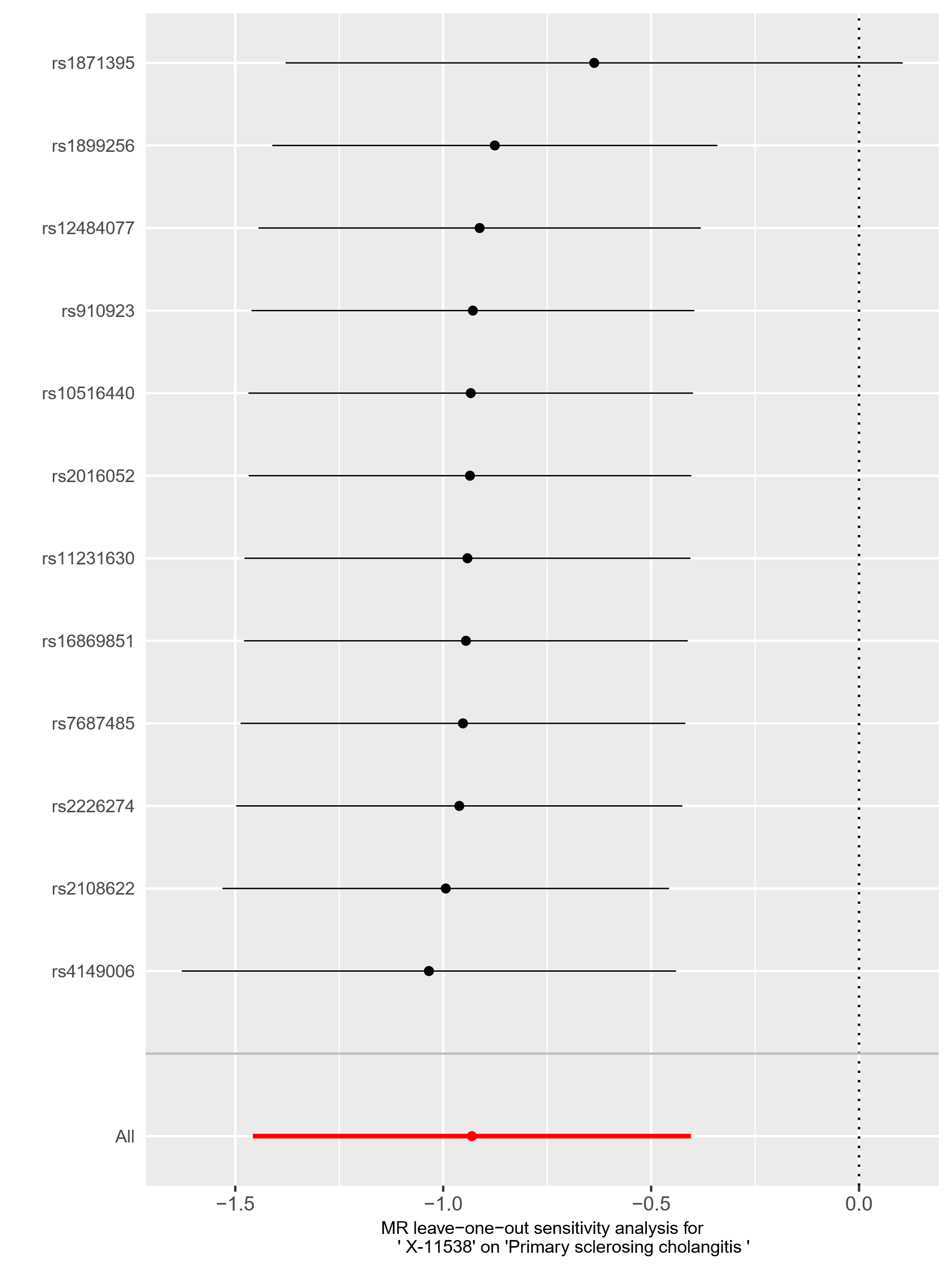

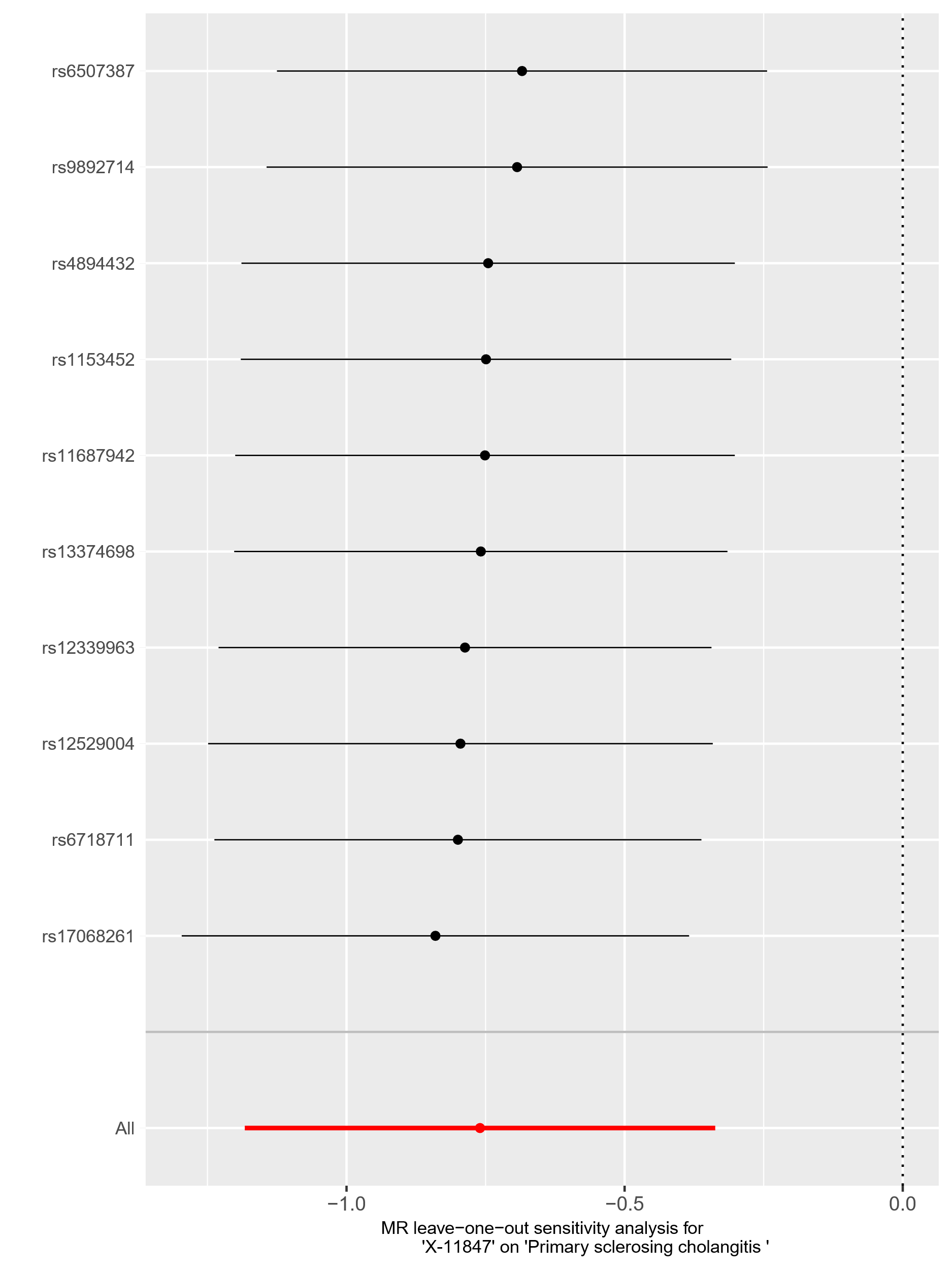

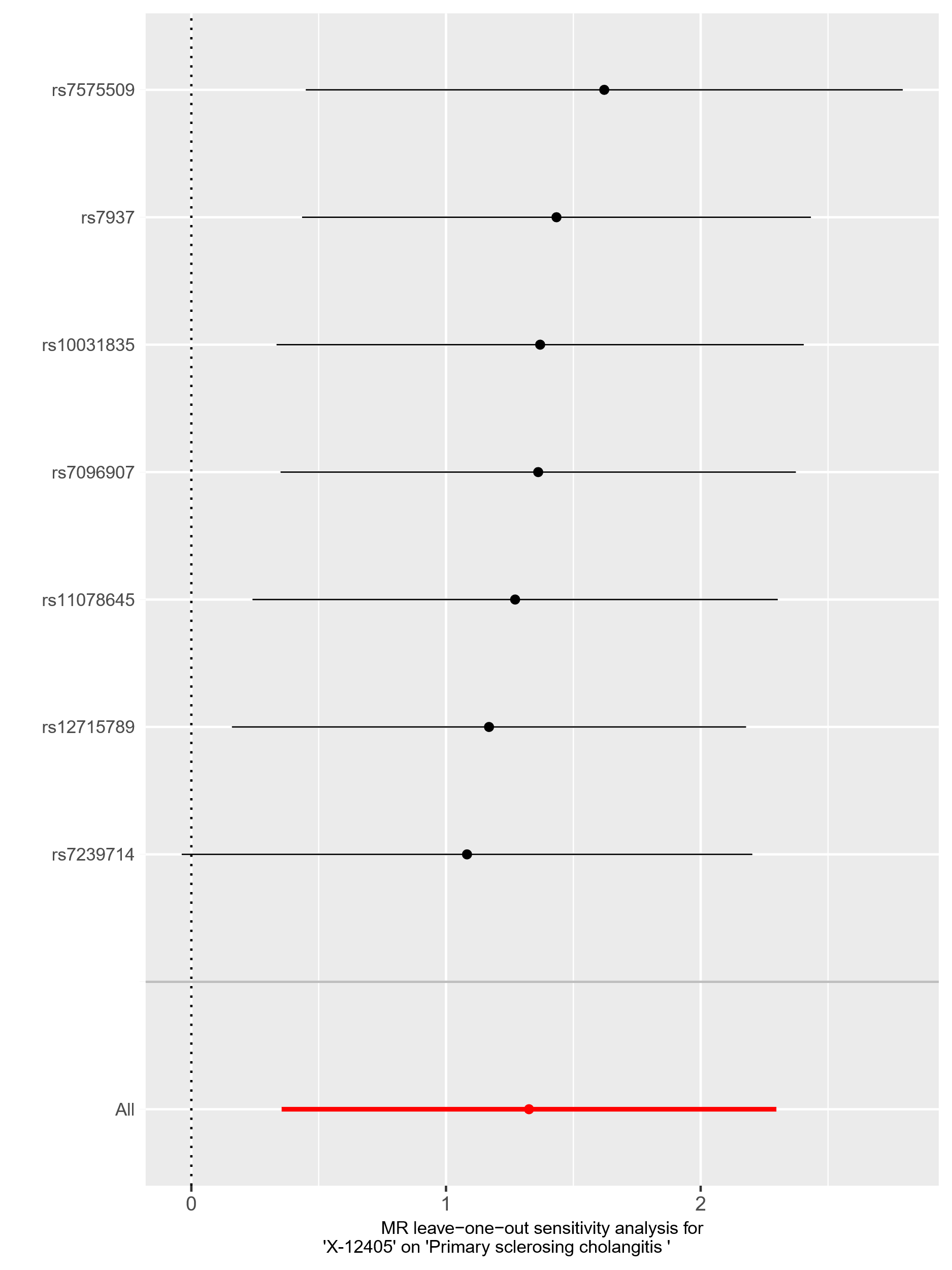

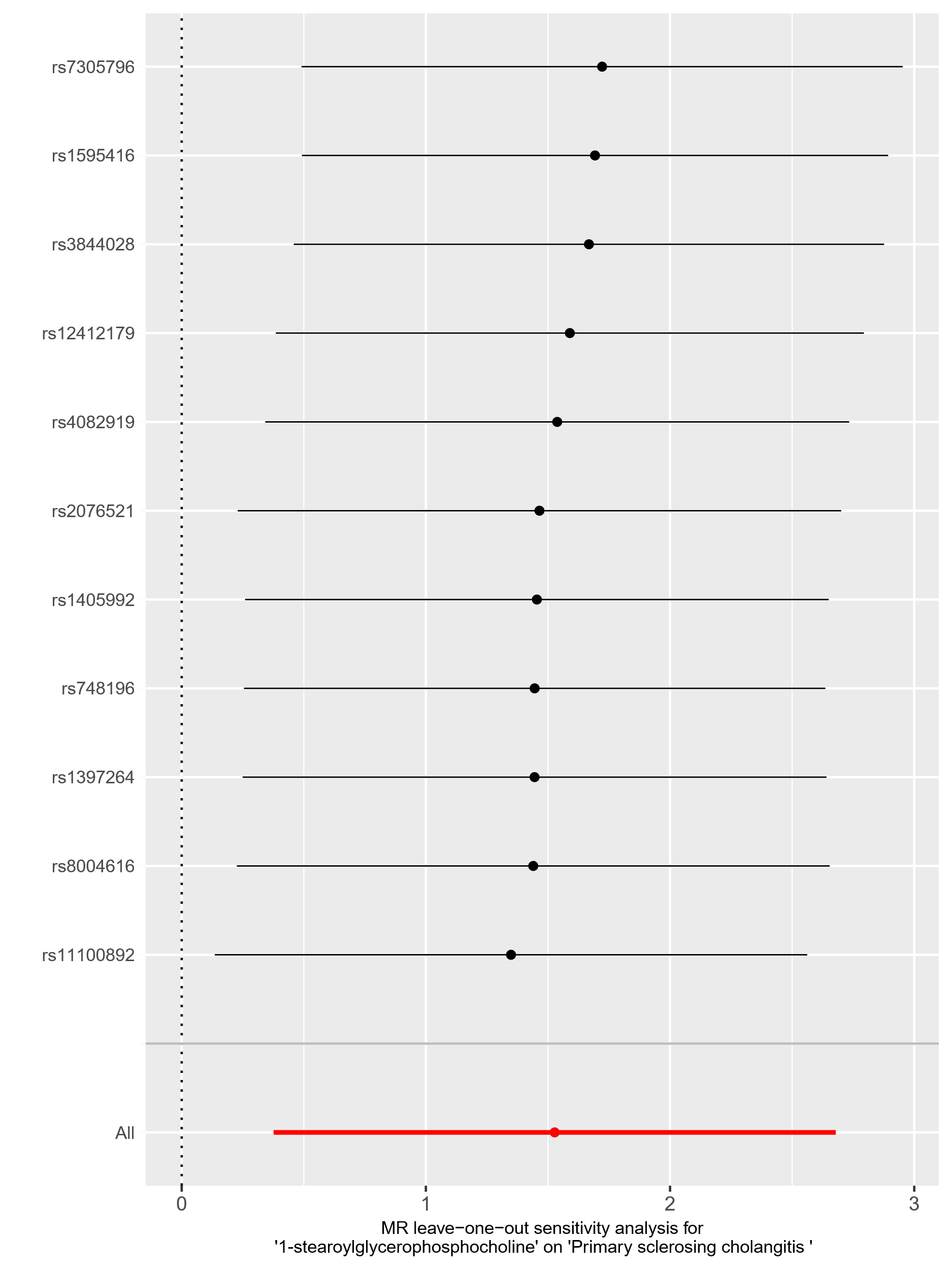

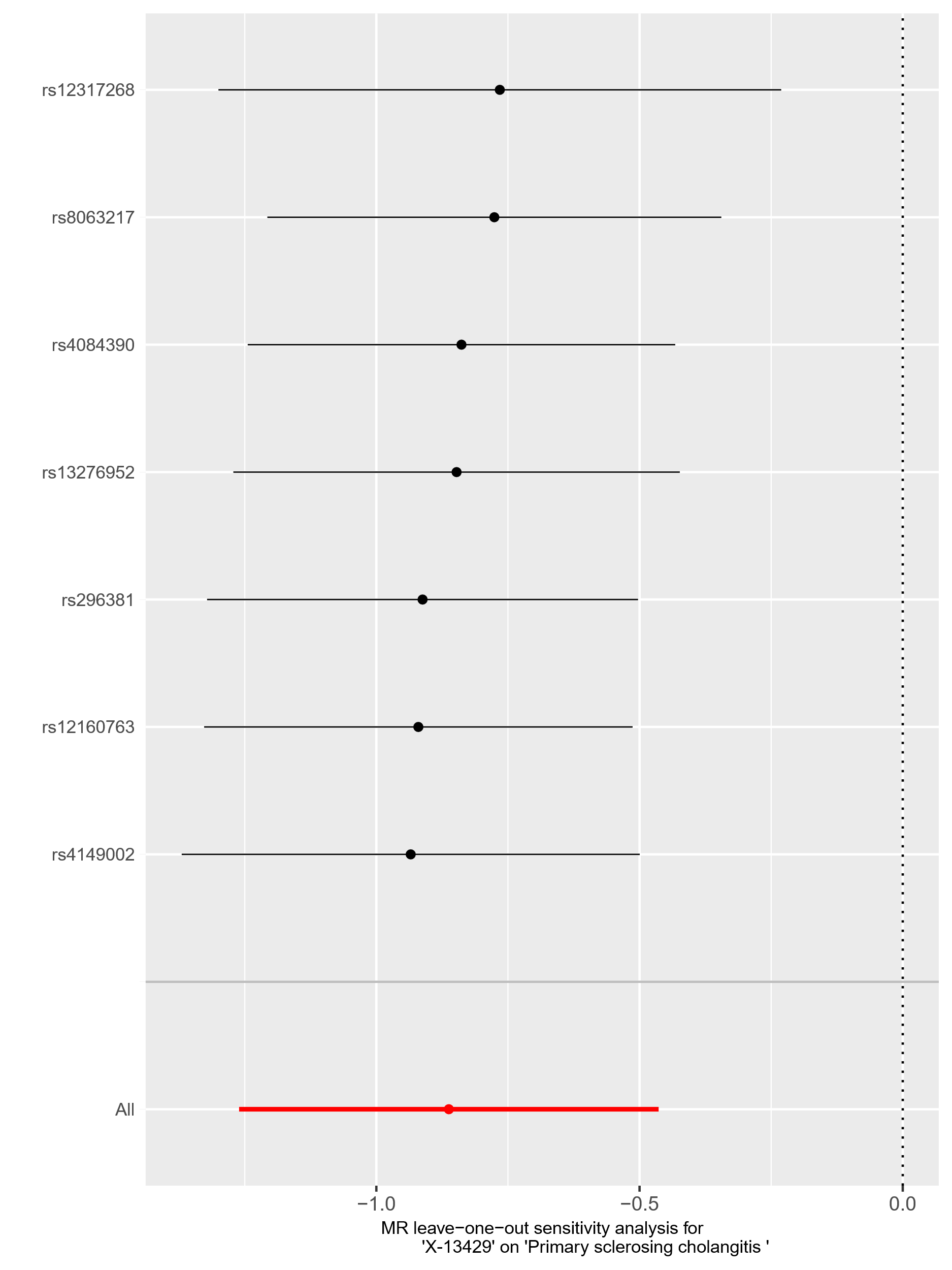


**Figure S2：** Forest plots for the Mendelian randomization (MR) leave-one-out analysis of the significant inverse variance weighted (IVW) estimates. single nucleotide polymorphism; PBC, primary biliary cholangitis; PSC, primary sclerosing cholangitis.
